# Immunoproteomic analysis of a Chikungunya poxvirus-based vaccine reveals high HLA class II immunoprevalence

**DOI:** 10.1371/journal.pntd.0007547

**Published:** 2019-07-05

**Authors:** Elena Lorente, Alejandro Barriga, Eilon Barnea, Concepción Palomo, Juan García-Arriaza, Carmen Mir, Mariano Esteban, Arie Admon, Daniel López

**Affiliations:** 1 Unidad de Presentación y Regulación Inmunes, Centro Nacional de Microbiología, Instituto de Salud Carlos III, Majadahonda (Madrid), Spain; 2 Department of Biology, Technion-Israel Institute of Technology, Haifa, Israel; 3 Department of Molecular and Cellular Biology, Centro Nacional de Biotecnología, Consejo Superior de Investigaciones Científicas (CSIC), Madrid, Spain; University of Texas Medical Branch, UNITED STATES

## Abstract

**Background:**

Efficient adaptive antiviral cellular and humoral immune responses require previous recognition of viral antigenic peptides bound to human leukocyte antigen (HLA) class I and II molecules, which are exposed on the surface of infected and antigen presenting cells, respectively. The HLA-restricted immune response to Chikungunya virus (CHIKV), a mosquito-borne *Alphavirus* of the *Togaviridae* family responsible for severe chronic polyarthralgia and polyarthritis, is largely unknown.

**Methodology/Principal findings:**

In this study, a high*-*throughput mass spectrometry analysis of complex HLA-bound peptide pools isolated from large amounts of human cells infected with a vaccinia virus (VACV) recombinant expressing CHIKV structural proteins was carried out. Twelve viral ligands from the CHIKV polyprotein naturally presented by different HLA-A, -B, and -C class I, and HLA-DR and -DP class II molecules were identified.

**Conclusions/Significance:**

The immunoprevalence of the HLA class II but not the HLA class I-restricted cellular immune response against the CHIKV structural polyprotein was greater than that against the VACV vector itself. In addition, most of the CHIKV HLA class I and II ligands detected by mass spectrometry are not conserved compared to its closely related O'nyong-nyong virus. These findings have clear implications for analysis of both cytotoxic and helper immune responses against CHIKV as well as for the future studies focused in the exacerbated T helper response linked to chronic musculoskeletal disorders in CHIKV patients.

## Author summary

Using a *high-throughput* immunopeptidomics analysis from human cells infected with a vaccine candidate and humanized mice models, the first twelve natural HLA ligands and epitopes from Chikungunya virus (CHIKV), a re-emerging pathogen, have been identified. The results reported reveal that the immunoprevalence of helper but not cytolytic immune response against CHIKV was greater than the virus vector. This has implications for immune response studies and for the analysis of the exacerbated helper response linked to chronic disorders in CHIKV patients.

## Introduction

Chikungunya virus (CHIKV) is a mosquito-borne virus, member of the *Alphavirus* genus of the *Togaviridae* family, causing acute febrile illness in affected people that can develop to chronic debilitating polyarthralgia and polyarthritis. This arboviral pathogen, that was identified in the former Tanganyika territory in 1952 [1,2], caused repeated epidemics in Africa and Asia from the 1960s–1980s [3,4]. The following decades were of relative inactivity, but CHIKV re-emerged in 2005 causing in the French overseas department of Reunion and other Indian Ocean islands a fiery epidemic with more than 700,000 patients and 250 deaths [4]. The next year, in another massive outbreak in India, several million people were affected by CHIKV [5]. In the last years, this virus has spread quickly [6], causing numerous outbreaks in both tropical and subtropical countries around the world with increasing severity [7,8]. In addition, several CHIKV outbreaks in Europe, mainly in Italy, have been reported recently [9]. Therefore, infection by this virus is a serious threat to global health, and CHIKV is at present considered a high priority emerging pathogen [10].

CHIKV is an enveloped virus with a positive-sense, 12kb single-stranded RNA genome that encodes two large polyproteins [11]. The first is the nonstructural P1234 precursor, where the C-terminal domain of the nonstructural protein 2 (nsP2) releases the four multifunctional nonstructural proteins. In contrast, in the maturation of the structural polyprotein three different proteases are involved: first, the capsid is autocatalytically released, and later the endoplasmic reticulum (ER) signal peptidase and furin proteases from the host generate 6K transmembrane and the three E1, E2, and E3 envelope proteins [11].

Although the immune mechanisms involved in the control of CHIKV disease are not fully characterized, CHIKV-infected humans show different timing of CD4^+^ and CD8^+^ T cell responses in the acute phase of infection. CHIKV-specific cytolytic CD8^+^ T lymphocyte-mediated immune response displays a peak at the first day post-illness onset [12], while the corresponding CD4^+^ T lymphocytes is increased around the end of the acute phase [12]. Abundant activated CD8^+^ T lymphocytes, responsible for destruction of the infected cells [13], can be detected in blood samples from patients two months postinfection [14]. In addition, the production of cytokines by CD4^+^ T helper lymphocytes stimulates the cell-mediated and antibody immune responses against CHIKV.

The human leukocyte antigen (HLA) class I and II antigen processing pathways are the key elements to trigger adequate antiviral CD8^+^ and CD4^+^ T lymphocyte functions, respectively. Proteolytic degradation by cytosol proteinases, mainly proteasomes, of the newly synthesized viral or cellular proteins, some of which are defective in synthesis or folding, generates peptides 8–10 residues long. These short peptides, after translocation to the ER lumen by transporters associated with antigen processing, bind to the newly synthesized HLA class I molecules. These stabilized HLA class I/peptide complexes are then exported to the surface of all nucleated cells for cytolytic CD8^+^ T cell recognition [15]. Moreover, antigen presenting cells synthesize HLA class II molecules that, after insertion in the ER, are later transported to endosomal compartments without binding antigenic peptides. Next, these organelles fuse with late endosomes, which can contain exogenous protein material as viral particles and/or extracellular host proteins that were previously engulfed by endocytosis, phagocytosis or pinocytosis. These exogenous proteins can be processed by the different lysosome-resident cathepsins, yielding cellular and viral peptides of different lengths by molecular mechanisms currently controversial (reviewed in [16]). These peptides, up to 30 residues long, stabilize the HLA class II molecules and then, these HLA class II/peptide complexes are transported to the cell membrane where they are exposed for CD4^+^ T helper cell recognition [17]. In absence of appropriate HLA class I and II-restricted T cell recognition both cellular and humoral immune responses cannot be efficiently activated and thus, the infective virus could spread within the whole organism with fatal results for the host.

The insertion of CHIKV structural genes inside the vaccinia virus (VACV) genome (rVACV-CHIKV) has been proposed as a vaccine candidate for this mosquito-borne virus, due to the high antiviral efficacy against CHIKV challenge [18]. Previously, the characterization of their immunogenicity profile and efficacy in the BALB/C mice model was carried out [18]. This recombinant virus was utilized, in addition to other different vaccine candidates including DNA-launched RNA replicon, soluble recombinant p62-E1 CHIKV protein and attenuated CHIKV, in several prime-boost protocols of immunization and virus challenge studies in a nonhuman primate model [19]. Moreover, to date, only one study using one of these vaccine candidates in humanized *in vitro* models has been carried out [20]. By selection of potential HLA class I ligands based on an HLA class I binding prediction algorithm [21], and subsequent analysis of peptide-specific IFNγ-secreting cells from rVACV-CHIKV-immunized HLA-A*0201 transgenic mice, three epitopes from the short (60 residues) 6K protein were identified [20]. However, no natural HLA class I or class II ligands for neither CHIKV natural infection nor CHIKV vaccine candidates has been described so far. In this work, using an immunopeptidomics analysis of HLA ligands which were isolated from large amounts of human cells infected with rVACV-CHIKV, we describe several natural CHIKV ligands and epitopes restricted by different common HLA class I and II molecules.

## Methods

### Mice and ethics statement

HLA-B*07:02 [22], and HLA-DRB1*04:04 [23] transgenic mice were bred in the animal facilities at Centro Nacional de Microbiología, Instituto de Salud Carlos III, in strict accordance with the recommendations in the Guide for the Care and Use of Laboratory Animals of the Spanish Comisión Nacional de Bioseguridad of the Ministerio de Medio Ambiente y Medio Rural y Marino (accreditation n° 28079-34A). The protocol was approved by the Research Ethics and Animal Welfare Committee of the Carlos III Health Institute (permit n°: PI-283). All surgery was performed under isoflurane anesthesia, and all efforts were made to minimize suffering.

### Cell lines

Two different Epstein–Barr virus (EBV)-immortalized B cell lymphoblastoid lines were used. The homozygous human HOM-2 cell line expresses HLA-A*03:01, -B*27:05, and -C*01:02 class I molecules and HLA-DR B1*01:01, and HLA-DP B1*04:01 chains. The human JY cell line expresses HLA-A*02:01, -B*07:02, and -C*07:02 class I molecules and four HLA-DR chains: B1*04:04, B4*01:01, B1*13:01, and B3*01:01 and two HLA-DP chains: B1*02:01, and B1*04:01. Both human cell lines were cultured in RPMI 1640 supplemented with 7% fetal bovine serum.

### Infection of the cell lines by VACV

The VACV Western Reserve strain expressing the CHIKV structural genes under control of a strong synthetic early/late virus promoter (termed rVACV-CHIKV), previously described [20], was utilized to infect 1x10^9^ HOM-2 or JY cells at a multiplicity of infection of 3 plaque-forming units/cell in 100 ml for 2 h at 37°C, and then cells were washed with PBS, as previously described [24]. These conditions were previously determined as the optimal to obtain infection of all cells without impairing the cell viability. Next, the cells were cultured for 4 h at 37°C and stained with the Omnitope antiserum-FITC that recognizes VACV virions (ViroStat Inc., Westbrook, ME, USA). Samples were analyzed measuring fluorescence intensity by flow cytometry to confirm VACV infection (1020 ± 87 mean fluorescence intensity [MFI] in VACV-infected cells versus 422 ± 48 in non-infected cells stained with the anti-VACV antiserum). The cells were then frozen in the presence of phenylmethanesulfonylfluoride (PMSF) until they were lysed.

### HLA-bound peptide isolation

**Fig 1:**
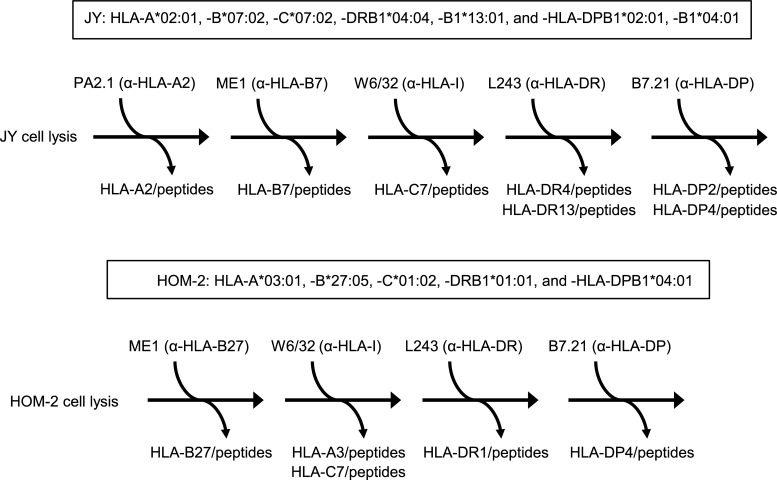
Diagram of the scheme of sequential immunoprecipitation. Uninfected or rVACV-CHIKV-infected JY or HOM-2 cells were lysed. HLA-peptide complexes were isolated via affinity chromatography of the soluble fraction of cell extracts with the following mAbs, sequentially used as indicated in the Fig: PA2.1 (anti-HLA-A*02), ME1 (anti-HLA-B*07, and -B*27), W6/32 (specific for a monomorphic pan-HLA class I determinant), L243 (HB55, specific for a monomorphic pan-HLA-DR determinant), and B7.21 (specific for monomorphic pan-HLA-DP determinant). The HLA class I and II molecules expressed in each human cell line is indicated in a box.

HLA-bound peptides were isolated from a total of 4 or 2x10^10^ uninfected or VACV-infected HOM-2 or JY cell lines for 6 hours, respectively. The cells were lysed in 1% CHAPS (Sigma), 20 mM Tris/HCl buffer, and 150 mM NaCl, pH 7.5 in the presence of the cOmplete, Mini Protease Inhibitor Cocktail (Merck KGaA, Darmstadt, Germany). After centrifugation, the supernatant was passed first through a control precolumn containing CNBr-activated Sepharose 4B (GE Healthcare, Buckinghamshire, UK) to remove non-specific peptides and proteins. Next, the HLA class I/peptide or HLA class II/peptide complexes were isolated sequentially via affinity chromatography from the soluble cell extract fraction with PA2.1 (anti-HLA-A*02) [25], ME1 (anti-HLA-B*07) [26], W6/32 (specific for a monomorphic pan-HLA class I determinant) [27], L243 (HB55, specific for monomorphic pan-HLA-DR determinant) [28], or B7.21 (specific for monomorphic pan-HLA-DP determinant) [29] monoclonal antibodies (mAbs), as shown in Fig 1. The HLA-bound peptides were eluted at 4°C with 0.1% aqueous trifluoroacetic acid (TFA), separated from the HLA molecules, and concentrated by ultra-filtration with a Centricon 3 filter (Amicon, Beverly, MA), as previously described [30].

### Electrospray-ion trap mass spectrometry analysis

Peptide mixtures recovered after the ultra-filtration step were concentrated using Micro-Tip reversed-phase columns (C_18_, 200 μl, Harvard Apparatus, Holliston, MA) [30]. Each C_18_ tip was equilibrated with 80% acetonitrile in 0.1% TFA, washed with 0.1% TFA, and then loaded with the peptide mixture. The tip was then washed with an additional volume of 0.1% TFA, and the peptides were subsequently eluted with 80% acetonitrile in 0.1% TFA. Lastly, the peptide samples were concentrated to approximately 20 μl using vacuum centrifugation [24,30].

The HLA class I or II peptides recovered from immunoprecipitated HLA specific mAbs, were analyzed by nanoLC-MS/MS using a Q-Exactive-Plus mass spectrometer that was fitted with an Ultimate 3000 RSLC nanocapillary UHPLC (Thermo Fisher Scientific, Waltham, MA), using the same parameters previously described [31]. The peptides were resolved on homemade 3.5 micron Reprosil C18-Aqua (Dr. Maisch GmbH, Ammerbuch-Entringen, Germany) capillary columns (75 micron ID and about 30 cm long [32] with a 5–28% acetonitrile linear gradient for 2 h in the presence of 0.1% formic acid at a flow rate of 0.15 μl/min. The dynamic exclusion was set to 20 sec and the automatic gain control value for the full MS was set to 3x10^6^. The selected masses were fragmented from the survey scan of mass-to-charge ratio (m/z) 300–1,800 AMU at resolution 70,000. The 10 most intense masses were selected for fragmentation by higher-energy collisional dissociation (HCD) from each full mass spectrum. No fragmentation was performed for peptides with unassigned precursor ion charge states or charge states of five and above. MS/MS spectra were acquired with a resolution of 17,500 at m/z 200. The target value of the MS/MS was set to 1x10^5^ and the isolation window to 1.8 m/z. The maximum injection time was set to 100 ms and normalized collision energy to 25 eV. The peptide match option was set to Preferred.

### Database searches

Raw mass spectrometry data were processed using Peaks 8.5 (Bioinformatics Solutions Inc., Waterloo, Canada) for peak-list generation from the nanoLC-MS/MS data. The peaks were identified using the Peaks 8.5 software programs using the human, VACV, and CHIKV parts of the UniProt/Swiss-Prot database (November 2017), which included 20267, 217, and 5 proteins, respectively. The search was not limited by enzymatic specificity and both the peptide mass and the fragment ion tolerances were set at 5 and 20 ppm, respectively. This search was not limited by any methodological bias (e.g., individual protein selection or HLA consensus scoring algorithm use). The following variable modifications were also analyzed: acetylation of N-terminal residue, cysteinilation (C), oxidation (M, P, H, F, Y, and W), methylation (N- and C-terminus) and phosphorylation (Y, S, and T). The identified peptides were selected when their -10logP score from Peaks 8.5 was > 21. In addition, the maximum false discovery rate (FDR) was set to 1%. No viral peptides were found in a search of the reversed database. When the MS/MS spectra fitted more than one peptide, only the highest scoring peptide was selected. The mass spectrometry data have been deposited to the MassIVE repository (https://massive.ucsd.edu) with the data set identifier MSV000082670.

### *In silico* binding prediction of HLA class I and II ligands

The predicted binding of each peptide to HLA class I molecules was calculated using the artificial neural network-based alignment method NetMHCpan (version 4.0) (available in http://www.cbs.dtu.dk/services/NetMHCIIpan/), and the MHC-I binding prediction from IEDB (available in http://tools.iedb.org/mhci/). Similarly, the predicted binding of each peptide to HLA-DR or -DP class II molecules was calculated using the artificial neural network-based alignment method NetMHCIIpan (version 3.2) (available in http://www.cbs.dtu.dk/services/NetMHCIIpan/), and the MHC-II binding prediction from IEDB (available in http://tools.iedb.org/mhcii/).

### HLA/peptide stability assays

The following synthetic peptides were used as controls in the HLA/complex stability assays: VACV A34_82-90_ (LPRPDTRHL, HLA-B*07-restricted) [33], and CMV pp65_7-15_ (RCPEMISVL, HLA-C*01-restricted) [34]. The RMA-S HLA-B*07-transfected cells were incubated at 26°C for 16 h. This allowed for empty HLA class I molecule expression (without any peptide) at the cell membrane that was stable at 26°C but not at 37°C. The cells were washed with FBS-free culture medium and incubated for 2 h at 26°C with various peptide concentrations in FBS-free medium. Cells were maintained at 37°C for additional 2 h incubation and then collected for flow cytometry analysis. This method allowed for the empty HLA class I molecules to become internalized, and thus we were able to discriminate between bound or unbound peptides. HLA expression levels were measured using the mAb ME1 (anti-HLA-B*07), as previously described [35]. Data were acquired on a FACSCanto flow cytometer (BD Biosciences, San Jose, CA, USA) and analyzed using BD FACSDiva software, version 6 (BD Bioscience). The cells that were incubated without peptides exhibited peak fluorescence intensities close to the background staining that were observed with the secondary mAb alone. The fluorescence index was calculated for each concentration point as the ratio of the mean peak channel fluorescence of the sample to that of the control incubated without peptide. Peptide binding was also expressed as EC_50_, which is the molar concentration of the peptide at 50% of the maximum fluorescence obtained in a concentration range of 0.01–200 μM.

### IFN-γ-secreting CD4^+^ and CD8^+^ T cell detection by ELISPOT

ELISPOT assays were performed as previously described [36] to detect antigen-specific T cell activation. Briefly, purified rat anti-mouse IFN-γ antibody (clone R4-6A2, BD Pharmingen, San Diego, CA, USA) was coated on 96-well MultiScreen HTS HA plates (Millipore, Billerica, MA, USA). The plates were incubated overnight at room temperature and were blocked with medium that was supplemented with 10% fetal bovine serum for 2 h at 37°C. Duplicate cultures of erythrocyte-depleted spleen cells were prepared from HLA class I or II-transgenic mice at 7 days (acute response) post intraperitoneal (i.p.) infection infection with 1x10^6^ pfu of sucrose-purified rVACV-CHIKV at different dilutions with 10^−5^ M peptide. The plates were incubated overnight at 37°C in a 5% CO_2_ atmosphere and were then washed with PBS-T (PBS 0.05% Tween-20). The plate wells were incubated for 2 h at room temperature with biotinylated anti-mouse IFN-γ mAb clone XMG1.2 (BD Pharmingen, San Diego, CA, USA), washed with PBS-T, and incubated for 1 h at room temperature with horseradish peroxidase-conjugated streptavidin. The plates were additionally washed before adding 3,3’-diaminobenzidine substrate (Sigma, St. Louis, MO, USA) in 50 mM Tris buffer pH 7.4 that contained 0.015% hydrogen peroxide. To enumerate the IFNγ responses, spots were counted and wells were photographed using a Leica EZ4 HD stereo microscope and LAS EZ software (Leica Microsystems, Germany). Additionally, the percentage of CD4^+^ or CD8^+^ T cells was determined by flow cytometry after staining spleen cells with FITC-conjugated anti-mouse CD4 (clone GK1.5, Miltenyi Biotec, Bergisch Gladbach, Germany) and CD8 (clone KT15, Proimmune, England, UK) antibodies, respectively. Events were acquired on a FACSCanto flow cytometer (BD Biosciences, San Jose, CA, USA) and analyzed using BD FACSDiva software, version 6 (BD Bioscience).

### Experimental design

Several previous analyses have shown that 2x10^10^ virus-infected human cells are enough to identify HLA-bound viral peptide pools [24,30,37–39]. A precolumn was utilized to remove non-specific binding proteins and peptides. Similar amounts of uninfected human cells were used as negative control to discriminate viral and cellular peptides (included in proteome databases as well as unknown peptides, whose parental proteins may not be included in current human databases) and to exclude erroneous assignments of viral peptides. In addition both human and VACV databases were used to identify non-CHIKV peptides. The CHIKV structural genes, inserted into the VACV genome, encode for 1248 amino acids. The proteome of VACV with 56795 residues is 40-fold greater than the CHIKV structural protein. Moreover, the human database utilized is 100-fold greater than the corresponding VACV database. Thus, the probability of erroneous CHIKV assignments is statistically insignificant. Also a maximum false discovery rate (FDR) of 1% was estimated by searching against the database with the reversed viral and human sequences. No viral peptides were found in the search of the reversed database utilized. Moreover, several synthetic peptides were purchased from Peptide 2.0 (Chantilly, VA, USA) and their MS/MS spectra were used to confirm the assigned sequences. A schematic representation of experimental design is shown in Fig 1. To analyze the statistical significance of the assays, non-parametric non-parametric Mann–Whitney U test was used. *P* values < 0.05 were considered to be statistically significant.

## Results

### Physiological processing in human rVACV-CHIKV-infected cells generates a canonical high affinity HLA-B*07:02 epitope from CHIKV capsid protein

Different HLA class I-bound peptide pools were isolated from large numbers of either uninfected or rVACV-CHIKV-infected cells from the human cell line JY (Fig 1). These peptide mixtures were subsequently separated by capillary reversed-phase HPLC and were analyzed on-line using tandem mass spectrometry. No CHIKV peptides were identified after immunoprecipitation of HLA-A*02:01 molecules. By means of bioinformatics tools, one fragmentation spectrum detected in the virus-infected HLA-B*07:02-bound peptide pools (but absent from control uninfected pool), was resolved with high confidence parameters as CHIKV structural polyprotein-derived peptide. This ion peak, with an *m/z* of 375.2, was assigned to the viral amino acid sequence RPWTPRPTI, spanning residues 17–25 of the capsid protein (CP) of CHIKV (Table 1 and S1 Fig). This theoretical assignment was confirmed by high similarity with the MS/MS spectrum of the corresponding synthetic peptide (S2 Fig). Additionally, a database search using human and VACV proteomes failed to identify this spectrum as human or VACV protein fragment, supporting the CHIKV origin of the fragment protein.

**Table 1:** Summary of the CHIKV ligands that were detected by MS/MS analysis in the rVACV-CHIKV infected cells.

Experimental mass ^a^	ΔMass ^b^	z	Sequence ^c^	Length	Protein ^d^	Position	HLA	Cell line	IEDB ^e^	NetMHCpan ^f^	IFNγ response
1122,630	-0.7	3+	RPWTPRPTI	9	CP	17–25	B*07:02	JY	0.2	0.01	+
1814,957	0.1	2+	YEHV**TVIPNTVGV**PYK	16	E1	1–16	C*07:02	JY	3.6	0.9	
1814,957	0.8	2+	YEHVTV**IPNTVGVPYK**	16	E1	1–16	A*03:01	HOM-2	3.55	3.27	
			YEHV**TVIPNTVGV**PYK	16	E1	1–16	C*01:02	HOM-2	0.69	0.37	
1518,842	3.1	4+	PPVIGREKFHSRP	13	E2	133–145	DRB1*04:04	JY	-	-	+
			PPVIGREKFHSRP	13	E2	133–145	DRB1*13:01	JY	0.27	0.23	
			PPVIGREKFHSRP	13	E2	133–145	DRB4*01:01	JY	-	-	
			PPVIGREKFHSRP	13	E2	133–145	DRB3*01:01	JY	-	-	
1296,698	0.5	4+	FKYWLKERGA	10	E1	240–249	DRB1*01:01	HOM-2	8	5	
1208,619	2.8	2+	RPGYYQLLQA	10	E3	44–53	DRB1*01:01	HOM-2	0.01	0.15	
1407,751	-3.7	2+	RPGYYQLLQASL	12	E3	44–55	DRB1*01:01	HOM-2	0.01	0.15	
1731,796	-2.6	3+	RPGYYQLLQASLTC*	14	E3	44–57	DRB1*01:01	HOM-2	0.01	0.15	
1915,881	-0.1	2+	RPGYYQLLQASLTC*SP	16	E3	44–59	DRB1*01:01	HOM-2	0.01	0.15	
2051,956	3.1	2+	RPGYYQLLQASLTC*SPH	17	E3	44–60	DRB1*01:01	HOM-2	0.01	0.15	
2208,057	1.1	4+	RPGYYQLLQASLTC*SPHR	18	E3	44–61	DRB1*01:01	HOM-2	0.01	0.15	
1814,957	4.3	2+	YEHVTVIPNTVGVPYK	16	E1	1–16	DPB1*04:01	HOM-2	-	-	
1020,539	3.5	2+	HPHEIILY	8	E2	351–358	DPB1*04:01	HOM-2	14	-	
1697,826	0.0	2+	IILYYYELYPTM*T	13	E2	355–367	DPB1*04:01	HOM-2	0.56	-	

^a^ The monoisotopic ion mass in amu.

^b^ The difference between nominal and experimentally detected monoisotopic ions in ppm.

^c^ Asterisks indicate oxidation of Met or cysteinylation of Cys. The minimal core for binding of E1 _1–16_ ligand to respective HLA class I molecule is bolded.

^d^ CP: capsid protein; E, envelope protein.

^e^ and ^f^ Theoretical affinity to HLA of the CHIKV ligands calculated as percentile rank from the Immuno Epitope Database (IEDB) or the NetMHCpan servers, respectively. (-), no binding.

**Fig 2:**
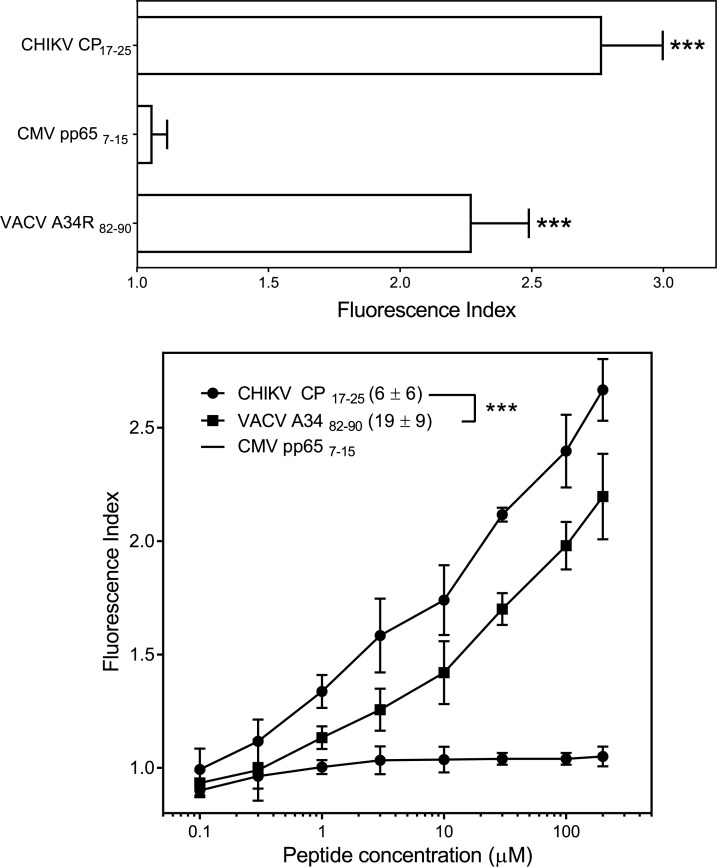
HLA-B*07:02 stabilization with CHIKV CP_17-25_ synthetic peptide. The stability of HLA-B*07:02-peptide complexes on the surface of transfected RMA-S cells was measured by flow cytometry. Top panel, the indicated peptides were used at 200 μM. The CMV pp65_7-15_ and VACV A34_82-90_ peptides were used as negative and positive controls, respectively. The mAb ME1 was used for staining. The results, calculated as fluorescence indexes, are shown as the mean ± SD of four independent experiments. Bottom panel, titration curves for the indicated synthetic peptides with HLA-B*07:02. Results are shown as mean values ± SD from four independent experiments. Calculated EC_50_ values (μM; mean ± SD) are shown in the left panel insert. Significant p values with a non-parametric Mann–Whitney U test: ***, p <0.001 versus negative peptide control (CMV pp65_7-15_, top panel) or between CHIKV CP_17-25_ and VACV A34_82-90_ viral peptides (bottom panel) are indicated.

The classical anchor motif for HLA-B*07:02 binding, Pro at position 2 (P2) and aliphatic C-terminal residues (SYFPEITHI database: http://www.syfpeithi.de [40]), were present in the CHIKV CP_17-25_ ligand. In addition, the prediction of peptide binding using two different computational approaches, the NetMHCpan neural network-based alignment method and the IEDB prediction software, suggested that this CHIKV peptide could be a HLA-B*07:02 high affinity ligand (Table 1). Later, HLA/peptide complex stability assays performed using TAP-deficient RMA-S cells transfected with the HLA-B*07:02 molecule showed that the CHIKV CP_17-25_ ligand bound to HLA-B*07:02 class I molecules with EC_50_ values in the range commonly found among other natural high affinity ligands, such as VACV A34_82-90_ (Fig 2).

**Fig 3:**
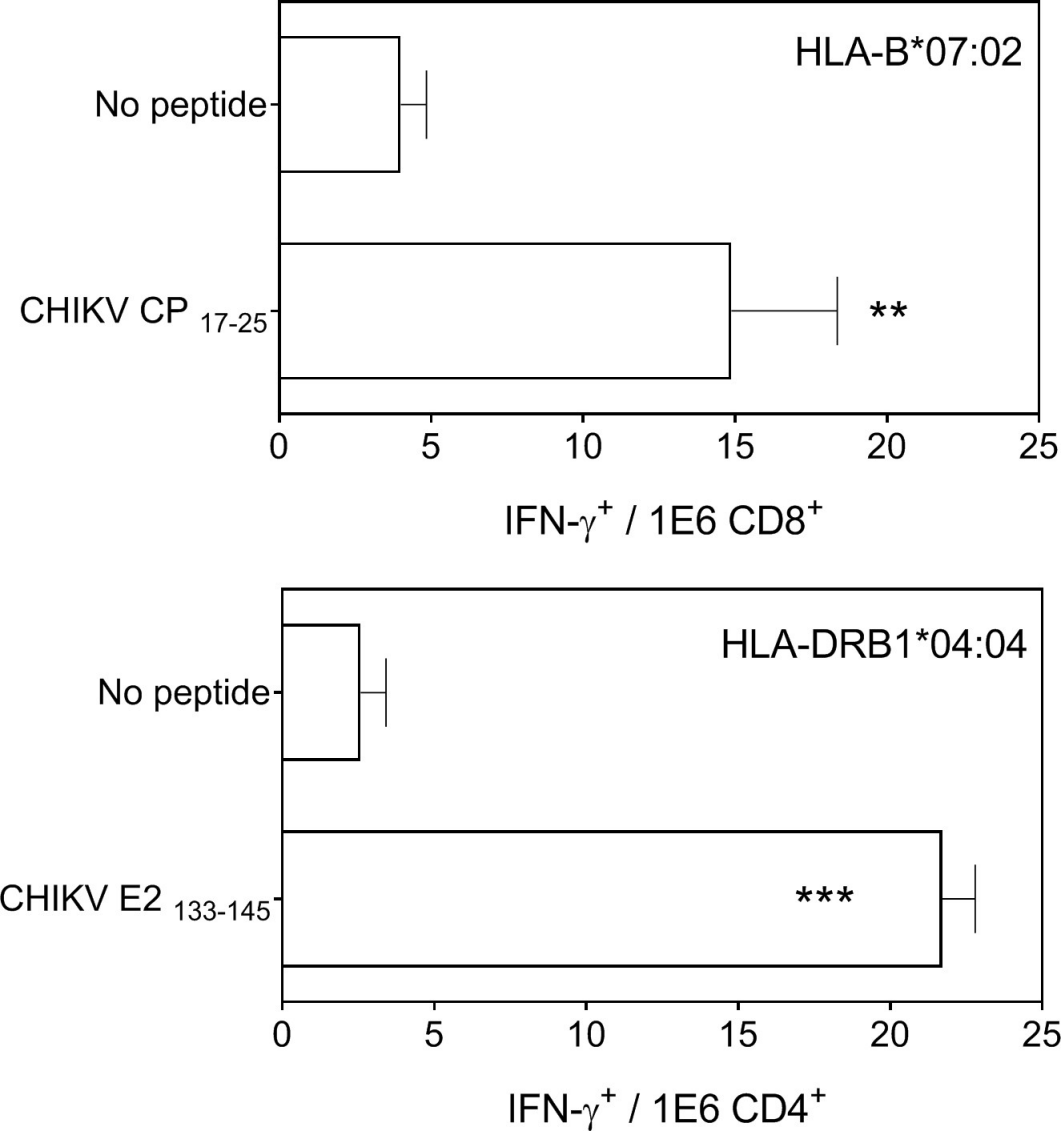
Immunogenicity of CHIKV-derived HLA-B*07:02, and DRB1*04:04-restricted peptides in HLA transgenic mice. HLA-B*07:02 (top panel), or -DRB1*04:04 (bottom panel) target cells that were pre-pulsed with the indicated CHIKV-synthetic peptides (CP_17-25_ and E2_133-145_) were analyzed by ELISPOT for CD8^+^ or CD4^+^ T cell activation with CHIKV-specific splenocytes obtained from HLA-B*07:02 (top panel), or -DRB1*04:04 (bottom panel) transgenic mice 7 days (acute response) post rVACV-CHIKV immunization (1x10^6^ pfu). The results are calculated as mean of seven independent experiments ± SD. Significant P values with a non-parametric Mann–Whitney U test: **, p <0.01; and ***, p <0.001 versus negative controls (no peptide) are indicated.

To study *in vivo* the physiological relevance of the identified CHIKV CP_17-25_ ligand, HLA class I-B*07:02 transgenic mice were infected by the i.p route for 7 days with rVACV-CHIKV. Later, a physiological measurement of the functional *ex vivo* activity of T cells against this viral ligand was carried out. Spleen cells of infected mice specifically recognized cells that were pulsed with the CHIKV CP_17-25_ peptide (Fig 3, upper panel).

Collectively, these results indicate that the CHIKV CP_17-25_ nonamer is a canonical high affinity HLA-B*07:02 epitope generated in the cellular immune response against the CHIKV structural polyprotein expressed from a rVACV-CHIKV vaccine candidate.

### An unusual CHIKV ligand was endogenously presented by HLA-C*07:02 class I molecules in human rVACV-CHIKV-infected cells

After sequential immunoprecipitation of HLA-A*02:01 and -B*07:02 class I molecules, the HLA-C*07:02-bound peptide pool was obtained from JY cells (Fig 1). One fragmentation spectrum from the rVACV-CHIKV-infected W6/32-bound pool, but absent in its control uninfected pool, was determined as a CHIKV-derived peptide (Table 1). Furthermore, the human and VACV proteome database searches also failed to identify this spectrum as a proteic fragment of these organisms, sustaining the CHIKV origin of this HLA-bound peptide. The ion peak, with an *m/z* of 908.5, was assigned to the viral amino acid sequence YEHVTVIPNTVGVPYK, which spans residues 1–16 of the CHIKV E1 envelope protein. S1 Fig shows the experimentally obtained MS/MS spectra and the respective assignments. In addition, the experimental fragmentation spectrum of CHIKV E1_1-16_ was absent in HLA-A*02:01 and -B*07:02 peptide pools, and thus this viral peptide was most likely a HLA-C*07:02 ligand.

HLA-C*07:02 class I molecules preferentially binds, as other HLA-A, -B, and -C class I proteins, peptides of 8 to 11 residues long (SYFPEITHI database [40]). The YEHVTVIPNTVGVPYK peptide is a 16mer and thus, must be a noncanonical HLA class I ligand. The MHC binding prediction by IEDB bioinformatic tool showed that the 11mer sequence TVIPNTVGVPY would bind with relatively high affinity to HLA-C*07:02 molecule (Table 1). Moreover, the 9mer with sequence TVIPNTVGV obtain a high affinity score using the NetMHCpan software for HLA-C*07:02 binding (Table 1). Thus, these binding prediction analyses suggest that the CHIKV E1_1-16_ peptide, identified by mass spectrometry, is an unusual HLA class I ligand that interacts with HLA-C*07:02 molecules by a central core and their N- and C-terminal residues probably protruded out of the HLA-C*07:02 binding groove.

### The CHIKV E1_1-16_ peptide was also presented by both HLA-A*03:01 and/or HLA-C*01:02 class I molecules in human rVACV-CHIKV-infected cells

Similarly to JY cells, sequential immunoprecipitation of HLA class I molecules was performed using the soluble fraction of cell extracts from the HOM-2 cell line (Fig 1). No CHIKV peptides were identified after immunoprecipitation of HLA-B*27:05 molecules. In contrast, the same fragmentation spectrum corresponding to CHIKV E1_1-16_ ligand, previously identified bound to HLA-C*07:02 was obtained from the rVACV-CHIKV-infected W6/32-bound pool of HOM-2 cells. As previous, this spectrum was absent in its control uninfected pool and the earlier immunoprecipitation of HLA-B*27:05 molecules (Table 1). Thus, the CHIKV E1_1-16_ peptide was endogenously processed and presented by HLA-A*03:01 and/or HLA-C*01:02 class I molecules in rVACV-CHIKV-infected cells.

Theoretical binding affinity and allele assignment of the CHIKV E1_1-16_ peptide was carried out by IEDB and NetMHCpan servers. Both computational approaches predict the 9mer sequence TVIPNTVGV (the same core for HLA-C*07:02 binding) as a high affinity ligand for HLA-C*01:02 class I molecules (Table 1). Moreover, using the same bioinformatic tools, the 10mer sequence IPNTVGVPYK ranks with a moderate score for binding prediction to HLA-A*03:01 class I molecule (Table 1). Thus, these results suggest that CHIKV E1_1-16_ peptide was most likely a non-canonical HLA-C*07:02 ligand, and even an HLA-A*03:01-bound peptide too.

### Multiple VACV ligands were naturally presented by six different HLA class I molecules in human rVACV-CHIKV-infected cells

Moreover, in addition to the three fragmentation spectra resolved as CHIKV structural polyprotein-derived peptides, other 69 fragmentation spectra present in any of the five virus-infected HLA class I-bound peptidic pools obtained (Fig 1), but absent in their respective control uninfected pool, were resolved with high confidence parameters as peptides derived from 40 different VACV proteins. In addition, the human proteome database searches also failed to identify these spectra as a human protein fragments, backing the viral origin of these HLA-bound peptides. These ion peaks were assigned to the VACV amino acid sequences described in Table 2. Thus, 6, 4, 12 and 15 peptide sequences were obtained bound to HLA-A*02:01, -B*07:02, -B*27:05, and -C*07:02 molecules respectively (Table 2). 36 of these 37 VACV peptides were predicted as ligands for the respective HLA class I molecule by one or both computational approaches utilized (Table 2). In contrast, IEDB and NetMHCpan algorithms failed to predict B*07:02-binding for the VACV A37_5-15_ ligand (Table 2). This peptide with Pro in P1, but not in P2 position as usual in B*07:02 ligands (SYFPEITHI database: http://www.syfpeithi.de [40]), would then be a non-canonical ligand for this HLA class I molecule.

**Table 2:** Summary of the VACV ligands that were detected by MS/MS analysis in the rVACV-CHIKV infected cells.

Experimental mass ^a^	ΔMass ^b^	z	Sequence ^c^^,^ ^d^	Length	Protein	Position	HLA ^d^	Cell line	IEDB ^e^	NetMHCpan ^f^
1041,659	2,7	2+	ALFGIKLPAL	10	A3	238–247	A*02:01	JY	0.44	0.39
998,674	-0,9	2+	SLLKSLLLL	9	A47	145–153	A*02:01	JY	0.7	0.17
1087,610	4	2+	YLLMHLVSL	9	B6	163–171	A*02:01	JY	0.2	0.91
1132,595	0	2+	KLFTHDIM*L	9	D12	62–70	A*02:01	JY	1	0.03
1070,674	-1	2+	LLFIPDIKL	9	K1	192–200	A*02:01	JY	3.4	0.19
970,654	-0,3	2+	KLVGKTVKV	9	K3	57–65	A*02:01	JY	1.3	0.03
1207,660	1,5	2+	PVFGISKISNF	11	A37	5–15	B*07:02	JY	-	-
1116,541	1,6	2+	SPRPTASSDSL	11	I3	12–22	B*07:02	JY	0.2	0.01
1049,707	-0,1	3+	LPRIALVRL	9	I8	227–235	B*07:02	JY	0.3	0.03
1029,622	0,9	2+	KPITYPKAL	9	K6	17–25	B*07:02	JY	0.4	0.18
1019,602	3,3	1+	YIIGNIKTV	9	A35	34–42	C*07:02	JY	-	0.54
1159,555	1,7	2+	NYVDYNIIF	9	B14	137–145	C*07:02	JY	0.38	0.08
1148,539	-0,3	1+	KVDDTFYYV	9	C7	74–82	C*07:02	JY	-	0.76
1198,646	0,6	2+	IPRSKDTHVF	10	D9	26–35	C*07:02	JY	3.2	2.57
1132,595	0	2+	KLFTHDIM*L	9	D12	62–70	C*07:02	JY	-	0.72
1174,602	-0,3	2+	RVYEALYYV	9	D12	251–259	C*07:02	JY	7.7	0.37
1183,671	0,8	3+	RIISYNPPPK	10	F8	56–65	C*07:02	JY	-	-
958,570	2,2	1+	SLKDVLVSV	9	G5.5	27–35	C*07:02	JY	-	0.15
1116,541	1,6	2+	SPRPTASSDSL	11	I3	12–22	C*07:02	JY	-	0.95
1087,555	1,6	1+	NLLENEFPL	9	K1	57–65	C*07:02	JY	2.87	0.06
1070,674	-1	2+	LLFIPDIKL	9	K1	192–200	C*07:02	JY	-	2.17
957,590	1,8	2+	LFIPDIKL	8	K1	193–200	C*07:02	JY	8.9	2.68
970,654	-0,3	2+	KLVGKTVKV	9	K3	57–65	C*07:02	JY	-	2.5
1029,622	0,9	2+	KPITYPKAL	9	K6	17–25	C*07:02	JY	-	0.97
1130,601	2,7	1+	YKPVYSYVL	9	N2	146–154	C*07:02	JY	6.6	0.48
1016,565	1	2+	GRLPLVSEF	9	A5	96–104	B*27:05	HOM-2	0.4	0.01
1038,593	-1,4	3+	NRTHAIISK	9	A51	228–236	B*27:05	HOM-2	1.1	0.45
1060,639	-1,7	3+	SRFANLIKI	9	A51	80–88	B*27:05	HOM-2	0.4	0.04
1192,708	1,4	3+	YRFLVINRL	9	B1	96–104	B*27:05	HOM-2	0.2	0.01
1174,646	1,1	2+	IRNDIRELF	9	C10	22–30	B*27:05	HOM-2	1.4	0.04
1202,591	1	3+	TRFYFNMPK	9	C10	323–330	B*27:05	HOM-2	0.4	0.3
1276,624	2,4	2+	GRFGYVPYVGY	11	D13	82–90	B*27:05	HOM-2	1.0	0.02
1096,534	1	2+	SRNPSKM*VY	9	E5	130–138	B*27:05	HOM-2	4.8	0.12
1173,687	-0,4	3+	IRILVEERF	9	E5	178–186	B*27:05	HOM-2	1.5	0.89
925,546	0,4	3+	GRIAPRSGL	9	F2	61–69	B*27:05	HOM-2	0.8	0.04
1321,704	-0,7	3+	HRFGLYRLNF	10	K2	121–128	B*27:05	HOM-2	0.28	0.04
1283,706	0,5	3+	GRLYKELMKF	10	K7	51–60	B*27:05	HOM-2	1.1	0.07
888,455	0	2+	GLAESSTPK	9	A4	10–18	A*03:01	HOM-2	0.34	0.12
			GLAESSTPK	9	A4	10–18	C*01:02	HOM-2	-	-
1107,676	0,1	3+	RLHEILTVK	9	A24	45–53	A*03:01	HOM-2	0.25	0.01
			RLHEILTVK	9	A24	45–53	C*01:02	HOM-2	-	-
1129,722	0,3	2+	IIIKKQFIQ	9	A24	758–766	A*03:01	HOM-2	-	4.7
			IIIKKQFIQ	9	A24	758–766	C*01:02	HOM-2	-	-
1212,713	-0,3	2+	KIFYKHIHK	9	A29	63–71	A*03:01	HOM-2	0.11	0.01
			KIFYKHIHK	9	A29	63–71	C*01:02	HOM-2	-	-
1332,693	1,6	2+	SVLC*VKKFYK	10	A34	159–168	A*03:01	HOM-2	0.21	0.2
			SVLC*VKKFYK	10	A34	159–168	C*01:02	HOM-2	-	-
1093,617	2,7	2+	KLFPVFTDK	9	B19	532–540	A*03:01	HOM-2	0.12	0.01
			KLFPVFTDK	9	B19	532–540	C*01:02	HOM-2	-	-
1182,695	1,8	3+	KVM*FVIRFK	9	C5	158–166	A*03:01	HOM-2	0.12	0.05
			KVM*FVIRFK	9	C5	158–166	C*01:02	HOM-2	-	-
948,564	0	2+	KVFDKSLL	8	D6	595–602	A*03:01	HOM-2	-	5.7
			KVFDKSLL	8	D6	595–602	C*01:02	HOM-2	1.8	1.18
1111,628	0,9	1+	KVFDKSLLY	9	D6	595–603	A*03:01	HOM-2	0.26	0.04
			KVFDKSLLY	9	D6	595–603	C*01:02	HOM-2	1.4	1.12
1239,723	0,2	3+	KVFDKSLLYK	10	D6	595–604	A*03:01	HOM-2	0.06	0.01
			KVFDKSLLYK	10	D6	595–604	C*01:02	HOM-2	-	-
1459,851	1,7	2+	VVA**DLSARNKLFK**	13	D12	99–111	A*03:01	HOM-2	0.34	1.32
			V**VADLSARNKL**FK	13	D12	99–111	C*01:02	HOM-2	2.3	-
963,550	-0,7	3+	SARN*KLFK	8	D12	104–111	A*03:01	HOM-2	5	5.2
			SARN*KLFK	8	D12	104–111	C*01:02	HOM-2	-	-
975,587	-0,4	3+	SLFKNVRL	8	D12	174–181	A*03:01	HOM-2	-	-
			SLFKNVRL	8	D12	174–181	C*01:02	HOM-2	3.1	2.3
1088,671	2,4	2+	SLFKNVRLL	9	D12	174–182	A*03:01	HOM-2	-	5.3
			SLFKNVRLL	9	D12	174–182	C*01:02	HOM-2	0.17	0.15
1216,766	0	2+	SLFKNVRLLK	10	D12	174–183	A*03:01	HOM-2	0.11	0.05
			SLFKNVRLLK	10	D12	174–183	C*01:02	HOM-2	-	-
1016,649	-2,6	3+	FKNVRLLK	8	D12	176–183	A*03:01	HOM-2	11.25	11.87
			FKNVRLLK	8	D12	176–183	C*01:02	HOM-2	-	-
1049,576	2	1+	KLFSDISAIG	10	E5	117–126	A*03:01	HOM-2	-	-
			KLFSDISAIG	10	E5	117–126	C*01:02	HOM-2	0.47	-
1177,671	0,6	1+	KLFSDISAIGK	11	E5	117–127	A*03:01	HOM-2	0.47	0.67
			KLFSDISAIGK	11	E5	117–127	C*01:02	HOM-2	5.58	0.01
789,423	0	2+	SDISAIGK	8	E5	120–127	A*03:01	HOM-2	-	11.17
			SDISAIGK	8	E5	120–127	C*01:02	HOM-2	-	-
1007,613	0,3	2+	TLNHVLALK	9	E6	32–40	A*03:01	HOM-2	0.13	0.03
			TLNHVLALK	9	E6	32–40	C*01:02	HOM-2	-	-
1113,629	-0,1	2+	KVYEAVLRH	9	E6	72–80	A*03:01	HOM-2	0.7	0.9
			KVYEAVLRH	9	E6	72–80	C*01:02	HOM-2	-	0.01
972,492	0,7	1+	ALYSYASAK	9	E9	677–685	A*03:01	HOM-2	0.11	0.01
			ALYSYASAK	9	E9	677–685	C*01:02	HOM-2	6.5	-
912,539	-0,6	2+	IAPRSGLSL	9	F2	63–71	A*03:01	HOM-2	-	8.8
			IAPRSGLSL	9	F2	63–71	C*01:02	HOM-2	0.02	8.6
1183,671	0,8	3+	RIISYNPPPK	10	F8	56–65	A*03:01	HOM-2	0.21	0.24
			RIISYNPPPK	10	F8	56–65	C*01:02	HOM-2	-	-
1015,607	1,5	2+	IVFNLPVSK	9	G8	65–73	A*03:01	HOM-2	0.18	0.01
			IVFNLPVSK	9	G8	65–73	C*01:02	HOM-2	9	9.3
991,618	0,2	2+	HIGIPISKK	9	H4	197–205	A*03:01	HOM-2	0.51	0.05
			HIGIPISKK	9	H4	197–205	C*01:02	HOM-2	-	-
1069,624	0,1	3+	TTPRKPAATK	10	H5	55–64	A*03:01	HOM-2	1.17	0.5
			TTPRKPAATK	10	H5	55–64	C*01:02	HOM-2	-	-
1028,577	0,1	2+	AVYGNIKHK	9	I3	116–124	A*03:01	HOM-2	0.3	0.01
			AVYGNIKHK	9	I3	116–124	C*01:02	HOM-2	-	-
911,544	1,6	2+	KVSPPSLGK	9	I8	72–80	A*03:01	HOM-2	0.13	0.01
			KVSPPSLGK	9	I8	72–80	C*01:02	HOM-2	-	-
1005,622	1,3	3+	TILKSLGFK	9	I8	239–247	A*03:01	HOM-2	0.17	0.12
			TILKSLGFK	9	I8	239–247	C*01:02	HOM-2	-	-
948,510	-0,3	1+	IIGPMFSGK	9	J2	9–17	A*03:01	HOM-2	0.4	0.1
			IIGPMFSGK	9	J2	9–17	C*01:02	HOM-2	-	-
1081,567	-0,7	2+	KIYEGAKHH	9	VACWR036	11–19	A*03:01	HOM-2	2.7	0.27
			KIYEGAKHH	9	VACWR036	11–19	C*01:02	HOM-2	-	-

^a^ The monoisotopic ion mass in amu.

^b^ The difference between nominal and experimentally detected monoisotopic ions in ppm.

^c^ Asterisks indicate oxidation of Met. The minimal core for binding of D12 _99–111_ ligand to respective HLA class I molecule is bolded.

^d^ HLA-A*03:01 and/or HLA-C*01:02 assignments (calculated as percentile rank < 5) are bolded.

^e^ and ^f^ Theoretical affinity to HLA of the CHIKV ligands calculated as percentile rank from the Immuno Epitope Database (IEDB) or the NetMHCpan servers, respectively. (-), no binding.

The other 32 sequences were obtained from the virus-infected W6/32-bound pool of the HOM-2 cells and thus, these peptides could be presented by HLA-A*03:01 and/or HLA-C*01:02 class I molecules in virus-infected cells. Theoretical binding affinity and allele assignment of these peptides was carried out by IEDB and NetMHCpan bioinformatics tools. 23 and 5 of these VACV sequences were assigned as HLA-A*03:01 and HLA-C*01:02-restricted ligands, respectively (Table 2). In addition, the other 4 VACV sequences (D6_595-603_, D12_99-111_, E5_117-127_, and E6_72-80_) of this peptide pool were assigned as viral ligands with theoretical affinity for both HLA-A*03:01 and -C*01:02 class I molecules (Table 2).

Thus, collectively these data indicate that all analyzed HLA class I molecules bound viral ligands. In addition, up to 73 different natural HLA class I-VACV peptide complexes (6 with HLA-A*02:01, 27 with HLA-A*03:01, 4 with HLA-B*07:02, 12 with HLA-B*27:05, 9 with HLA-C*01:02, and 15 with HLA-C*07:02) can be presented in the two virus-infected cell lines.

### The immunoprevalence of the HLA class I-restricted cellular immune response against CHIKV structural polyprotein was similar to that against the VACV vector

In this study, four different natural HLA class I-CHIKV peptide complexes, each one restricted by the HLA class I molecules HLA-A*03:01, HLA-B*07:02, HLA-C*01:02, and HLA-C*07:02, were found (Table 1). The CHIKV structural genes, inserted into the TK locus of the VACV genome, encode for 1248 amino acids. Thus, the ratio was one HLA class I-CHIKV peptide complexes per 312 residues. Similarly, we found other 73 natural HLA class I-VACV peptide complexes and the proteome of the WR strain of VACV contains 56795 residues and thus, their ratio was one HLA class I-VACV peptide complexes per 778 residues. Although the ratio is a little more than double in CHIKV than in VACV proteins, however this difference was not statistically significant. Moreover, if only the two different HLA class I CHIKV ligands detected by mass spectrometry are considered, the CHIKV ratio was one HLA class I Iigand per 624 residues. Similarly, with the 69 fragmentation spectra from VACV proteome the equivalent ratio was one HLA class I-VACV ligand per 823 residues. Thus, the ligands to which HLA class I antigen processing and presentation are addressed to, that is, the immunoprevalence of the HLA class I-restricted cellular immune response against CHIKV structural polyprotein was comparable with a well-established poxvirus vaccine.

### One CHIKV ligand was presented by HLA-DRB1*04:04 class II molecules in human rVACV-CHIKV-infected cells and recognized by specific T cells in virus-infected HLA-DRB1*0404 transgenic mice

Similarly to HLA class I, the HLA-DR-bound peptide pools were isolated from either uninfected or VACV-infected JY cells. The ion peak with an *m/z* of 381.0, absent in the HLA-DR-uninfected pool, was assigned to the viral amino acid sequence PPVIGREKFHSRP, which spans residues 133–145 of the CHIKV E2 envelope protein. (Table 1 and S1 Fig). Furthermore, the database searches with human and VACV proteome also failed to identify this mass spectrometry spectrum, supporting the CHIKV origin of this HLA class II-bound peptide. This theoretical assignment was confirmed by identity with the MS/MS spectrum of the corresponding synthetic peptide (S3 Fig). The JY cell line expresses four HLA-DRB1 chains: B1*04:04, B1*13:01, B3*01:01 and B4*01:01. Thus, prediction analysis of HLA binding of the CHIKV E2_133-145_ ligand was carried out. The two bioinformatics methods utilized predicted high affinity values for DRB1*13:01 chain binding but not for other three HLA-DR chains from JY cell line (Table 1). As at the present day, these bioinformatics tools are not totally accurate in their predictions, especially to detect the non-canonical ligands, HLA-DRB1*04:04 transgenic mice were infected with rVACV-CHIKV; other HLA class II-transgenic mice were not available. Later, a physiological measurement of the functional *ex vivo* activity of T cells against the CHIKV E2_133-145_ ligand was carried out. Spleen cells of immunized mice specifically recognized cells pulsed with this peptide as part of the acute response to the vaccine utilized (Fig 3, bottom panel).

In summary, the CHIKV E2_133-145_ protein fragment was presented and recognized as an epitope bound to DRB1*04:04, and probably could be presented by DRB1*13:01 chain as well.

### Physiological processing generated seven CHIKV HLA-DRB1*01:01 ligands in human rVACV-CHIKV-infected cells

Similarly to JY cell line, the HLA-DR-bound peptide pools were isolated from either uninfected or rVACV-CHIKV-infected HOM-2 cells (Fig 1). In this experiment, seven different fragmentation spectra presented in the virus-infected HLA-bound peptide pool, but absent from the control uninfected pool, were determined as CHIKV E1, and E3 protein peptides (Table 1 and S1 Fig). Additionally, both human and VACV proteome database searches failed to identify any of these spectra, consistent with the CHIKV origin of these peptides.

The ion peak, with an *m/z* of 325.18 was assigned to the viral sequence FKYWLKERGA, which spans residues 240–249 of the CHIKV E1 protein (Table 1 and S1 Fig). In addition a nested set of six viral ligands with the same core and various C-terminally extended residues were found. The first ion peak, with an *m/z* of 605.31, was assigned to the viral 10-mer sequence RPGYYQLLQA, which spans residues 44–53 of the CHIKV E3 protein (Table 1 and S1 Fig). Five additional ion peaks with *m/z* of 704.88, 578.27, 958.94, 1026.98, and 553.02 were assigned to different viral amino acid sequences of the same CHIKV E3 protein that included the 44–53 minimal core with 2, 4, 6, 7, and 8 C-terminally extended residues, respectively (Table 1 and S1 Fig).

The homozygous HOM-2 cell line expresses HLA-DR B1*0101 chains. Both IEDB and NetMHCpan algorithms predicted high affinity binding to this HLA-DR chain for the six peptides included in the nested set of CHIKV E3 ligands identified by mass spectrometry (Table 1). The CHIKV E1_240-249_ peptide showed more moderate affinity values with both bioinformatics tools (Table 1).

### Three HLA-DPB1*04:01 ligands were generated in rVACV-CHIKV-infected cells

In addition, the HLA-DP-bound peptide pools were also isolated from either uninfected or rVACV-CHIKV-infected JY and HOM-2 cell lines (Fig 1). No CHIKV peptides were identified after immunoprecipitation of HLA-DP molecules from JY cells. In contrast, from the HOM-2 cells three different fragmentation spectra presented in the virus-infected HLA-DP-bound peptide pool but absent from the control uninfected pool, were determined as CHIKV E1, and E2 protein peptides (Table 1 and S1 Fig). Additionally, no human or VACV sequences could be assigned to these MS/MS spectra using proteome database searches.

The ion peak, with an *m/z* of 908.48 was assigned to the CHIKV E1_1-16_ peptide, the same previously identified bound to HLA-A*03:01, HLA-C*01:02, and HLA-C*07:02 (Table 1 and S1 Fig). In addition, the ion peaks with *m/z* of 511.00 and 849.92 were assigned to the partially overlapping sequences HPHEIILY and IILYYYELYPTMT, which spans residues 351–358 and 355–367 of the CHIKV E2 protein (Table 1 and S1 Fig). The homozygous HOM-2 cell line expresses HLA-DPB1*04:01 chains. IEDB, but not NetMHCpan, algorithm predicted high affinity binding to this HLA-DP chain only for the CHIKV E2_351-358_ peptide, and very low score for the CHIKV E2_355-367_ peptide (Table 1).

### The immunoprevalence of the HLA class II-restricted cellular immune response against CHIKV structural polyprotein was greater than for the VACV vector

In this study, 12 different natural HLA class II-CHIKV peptide complexes, restricted by four HLA class II molecules, were identified (Table 1). As the CHIKV structural genes encode for 1248 residues, thus, the ratio was one HLA class II-CHIKV peptide complex per 104 residues.

Likewise, 30 natural HLA class II-VACV peptide complexes were previously identified in the same rVACV-CHIKV-infected cells [41]. The source of ligands naturally bound to the HLA class II molecules is mostly derived from proteins included in the viral particles and/or from the extracellular medium, which were previously engulfed by endocytosis, phagocytosis, or pinocytosis, and later processed by several resident cathepsins from lysosomes. Thus, about half of the VACV WR strain proteome may be susceptible to entry in the HLA class II antigen processing and presentation pathways according to the information available in the UniProtKB database (https://www.uniprot.org/help/uniprotkb). 30290 residues are included into these 97 viral proteins and thus, the ligand/residues ratio was one HLA class II-VACV peptide complex per 1010 residues. Moreover, if only the six different HLA class II core CHIKV ligands detected by mass spectrometry are considered, the CHIKV ratio was one HLA class II Iigand per 208 residues. Similarly, with the 23 different HLA class II core ligands from VACV proteome the equivalent ratio was one HLA class II-VACV ligand per 1317 residues. These six or ten-fold enrichments in HLA class II-restricted ligands from CHIKV proteins versus the respective VACV proteins, that were statistically significant (P value < 0.001, Fisher's exact test), indicate a high immunoprevalence of the HLA class II-restricted cellular immune response against CHIKV structural polyprotein in the vaccine construct.

### Most of CHIKV HLA class I and II ligands detected by mass spectrometry are not conserved compared to O'nyong-nyong virus

O'nyong-nyong virus is closely related to CHIKV, as long as mAbs specific for CHIKV recognized most of O'nyong-nyong virus antigenic sites, albeit mAbs against O'nyong-nyong virus do not recognized the CHIKV particles [42]. Thus, to evaluate the possible cross-reactivity at cellular immune response level, an amino acid sequence comparison was carried out for the HLA class I and II ligands, identified by mass spectrometry, from CHIKV with the representative Igbo Ora strain of O'nyong-nyong virus. Table 3 shows that only the E2_351-358_ ligand sequence was conserved between both related alphaviruses. In addition, although the E1_1-16_ ligand presents a change in P4 position versus the respective O'nyong-nyong virus sequence, this mutation could not alter the predicted binding of this peptide to HLA class I molecules as suggest from the bioinformatic tools (Tables 1 and 3). In summary, the HLA molecules mainly binds not conserved peptides of CHIKV compared to O'nyong-nyong virus, in accordance with the 86% of amino acid identity of proteins between both virus. Thus, this analysis suggests that the crossreactivity of CHIKV HLA ligands from rVACV-CHIKV against homologous proteins from O'nyong-nyong virus would be unlikely.

**Table 3:** Conservation of CHIKV HLA viral ligands in *O'nyong-nyong virus*.

Virus ^a^	CP _17–25_ ^b^ (B*07:02)	E1 _1–16_ ^c^ (A*03:01, C*01:02, C*07:02, DPB1*04:01)	E1 _240–249_ (DRB1*01:01)	E2 _133–145_ (DRB1*04:04, DRB1*13:01)	E2 _351–358_ (DPB1*04:01)	E2 _355–367_ (DPB1*04:01)	E3 _44–61_ (DRB1*01:01)
CHIKV	RPWTPRPTI	YEHV**TVIPNTVGVPYK**	FKYWLKERGA	PPVIGREKFHSRP	HPHEIILY	IILYYYELYPTMT	RPGYYQLLQASLTCSPHR
*O'nyong-nyong*	----Q----	---A------------	-------K--	--L----------	--------	-----------T-	Q-------DSA-A--Q--

^**a**^ *Igbo Ora* strain is representative of *O'nyong-nyong* virus.

^**b**^ Protein and position of each ligand and in parenthesis the respective HLA presenting molecule.

^**c**^ The residues predicted by informatics tools as HLA ligands are showed in bold.

## Discussion

Different issues about the nature of the HLA class I and II-specific response against CHIKV can be derived from the results reported here.

Natural HLA class I ligands have mostly 9–11 residues long and display the canonical anchor residues (usually at position 2 and the C-terminus of the peptide ligand) for specific MHC binding (SYFPEITHI database [40]). These canonical peptides include the CHIKV CP_17-25_ epitope restricted by HLA-B*07:02 and 68 of the 69 VACV HLA class I ligands identified in the current report (Tables 1 and 2). Two exceptions were found: the CHIKV E1_1-16_ ligand identified both JY and HOM-2 cells, and the VACV D12_99-111_ ligand from HOM-2 cell line with 16 and 13 amino acid long, respectively. The existence of these viral ligands, with bioinfomatically predicted 9 or 10 canonical cores for binding to their respective HLA class I molecules, and N- and/or C-terminally extended residues that probably protruded out of the corresponding HLA class I binding groove as described in other non-canonical HLA class I ligands [43,44], can be explained for absence of the peptidase trimming. Usually, long peptides generated by proteasome and other cytosolic proteases are trimmed by ERAP1 and/or ERAP2 aminopeptidases in the ER to 9–11 mer ligands [45]. ERAP1 binds long substrates for both terminal residues, where the lateral chain of the C-terminus residue interacts with a hydrophobic pocket away from the active site. Thus, the N-terminal residue is accessible to the active site and then, this enzyme trims the longer precursors in a nonprocessive manner: the "molecular ruler" mechanism [46] [47]. The presence of basic residues in the C-terminus of peptide substrate prevents their interaction with ERAP1, and thus, removal of N-terminal residues is abolished [46]. In both CHIKV E1_1-16_ and VACV D12_99-111_ ligands the C-terminal residue is a Lys and thus, these long peptides are protected from ERAP1 activity. In contrast, this hydrophobic pocket is absent in the ERAP2 protease and thus, the C-terminal residue of substrate is not relevant for the activity of this proteinase [48]. Nevertheless, the high preference of ERAP2 for some amino acids in the N-terminal position, among which the Val and the Tyr amino acids are not included [49,50], would explain the presence of CHIKV E1_1-16_ and VACV D12_99-111_ ligands in virus infected cells. In addition, this absence of aminopeptidase trimming over these two long viral ligands would allow the formation of five different HLA-peptide complexes in the cell surface of infected cells, if the theoretical binding prediction by bioinformatics tools showed in Tables 1 and 2 is accurate. In contrast, if these long ligands would have been substrates of ERAP1 and/or ERAP2 proteases, the resulting 9-11mer products of trimming could only have bound to HLA-A*03:01 molecules since the anchor motifs at position P2 for HLA-C*01:02 and -C*07:02 would lack the binding. Thus, absence of aminopeptidase activities could, in some cases, increase the repertoire of HLA class I-peptide complexes exposed to T cell recognition in the surface of the infected cell. These data reinforce the need to identify the natural ligands versus other experimental strategies as is the analysis with synthetic peptides.

Human MHC ligands from CHIKV are largely unknown, since only three 6K transmembrane protein-derived epitopes presented by the HLA class I molecule A*02:01 were previously identified [20]. In the current report, using a high throughput immunoproteomic analysis from human cells infected with a rVACV-CHIKV vaccine expressing the CHIKV C, E3, E2, 6K, and E1 structural genes we identified several CHIKV ligands and epitopes associated to different HLA-A, -B, and -C class I molecules and HLA-DR, and -DP class II molecules, that derived from the capsid protein and the three envelope viral proteins (E1, E2, and E3), featuring the basis of both HLA class I and II antigen processing that trigger both cytotoxic and helper T cell responses against this reemerging virus. In addition, in this report the immunoprevalence of the proteins encoded by the CHIKV gene inserted in the recombinant VACV WR strain versus the VACV parental vector was also compared. In HLA class I, the abundance (relative to length protein) of CHIKV ligands was comparable in numbers with the one obtained from VACV proteins. In contrast, an enrichment in HLA class II-restricted ligands from CHIKV structural proteins versus the respective VACV proteins was found and thus, this high immunoprevalence of the HLA class II-restricted cellular immune response against CHIKV ligands (a key element to trigger protective both cellular and humoral immune responses) could mean that recombinant poxvirus-based vaccines against CHIKV could be effective.

Although a robust T cell response against infection is generally desirable, a major issue must be considered concerning CHIKV-related pathology. Vigorous adaptive immune responses in chronic CHIKV patients who displayed higher viral loads during the acute phase were previously found [51]. Remarkably, infiltrating CD4^+^ but not CD8^+^ T cells were found in the injured synovial tissue of these chronic CHIKV patients [51], suggesting a role of these lymphocytes in the chronic arthralgia/arthritis. More specifically, in the mice model these CHIKV-specific CD4^+^ but not CD8^+^ T lymphocytes were strongly associated to the inflammation in the joints by an IFN-γ-independent mechanism [52]. In chronic disease, CHIKV RNA and proteins are detected in musculoskeletal tissue, indicating that the immune response of the host fails to eliminate the pathogen from some tissues [53]. In addition, transfer of splenic CD4^+^ T lymphocytes from CHIKV-infected wild-type mice generated a severe joint inflammation into recipient animals of these HLA class II-restricted T helper cells [54]. Thus, an exacerbated T helper response causing chronic arthralgia and/or arthritis by the high immunoprevalence of the HLA class II-restricted cellular immune response against CHIKV structural polyprotein in the vaccine construct should be assessed and eventually ruled out in later studies. However, the depletion of CD4^+^, but not CD8^+^ T cells, after vaccination with an MVA-CHIKV vaccine expressing E2 and E3 CHIKV proteins in mice that were later infected with CHIKV caused death of all animals analyzed, demonstrating the indispensable role of CD4^+^ T lymphocytes in the protection [55]. Although MHC class II^-/-^ mice are able to control CHIKV viraemia, these MHC class II-deficient mice have 2.5 logs higher than normal mice with a delay in viraemia control [56]. Thus, it is possible that specific CD4^+^ T cells induced by MVA-CHIKV can help to effectively control the viral replication of CHIKV while minimizing collateral tissue injury.

Antibody-mediated immune response against CHIKV seems to mainly target the envelope glycoprotein E2 of CHIKV, although antibodies against E3, capsid and nsP3 proteins were also detected during the course of the disease [57]. In addition, this humoral early immune response is dominated by IgG3 antibodies specific mostly for a single linear epitope in the N-terminus of the viral E2 protein [58]. Although the sequences of the B and T cell epitopes are different, the immunoprevalence of the HLA class II ligands identified in the current report were also mainly focused on the E2 and E3 proteins.

Despite our data support a highly immunogenic vaccine, according to the very limited conservation between the HLA class I and II ligands from CHIKV proteins and their corresponding sequences in O'nyong-nyong virus, it is unlikely that the recombinant CHIKV vaccine may have some kind of cross-protective cellular immune response against this second alphavirus although mAbs specific for CHIKV recognized most of O'nyong-nyong virus antigenic sites [42]. Our results also suggest that studies evaluating class I and class II responses against alphaviruses should be carried out individually due to the scarce conservation between epitopes in different virus species.

To explain the generation of HLA class II ligands, two different models have been proposed. The first, the “cut/trim first, bind later” model, suggests that the proteolytic activity of lysosomal proteases on the viral protein generates peptides of different length and later, these protein fragments bind to HLA class II molecules. Conversely, the “bind first, cut/trim later” model assumes that first the HLA class II molecules interact with the exposed regions from viral proteins, and later the lysosomal proteases trim the unprotected protruding ends of antigenic proteins to release the corresponding HLA class II/peptide complex. Some studies supporting each of the two models are reviewed in [16]. For example, a recent study has shown that different HLA class II-restricted epitopes from Zika virus are in outer regions of the virus envelope proteins [59] and thus, exposed to allow the direct interaction with HLA class II molecules as suggest the “bind first, cut/trim later” model. In contrast, the X-ray crystallography [60] shows that all HLA-DR and -DP class II ligands from CHIKV E1 and E2 envelope proteins identified in the current study are located in the internal regions of these viral proteins and thus, their generation would be compatible with the “cut/trim first, bind later” model. Alternatively, the CHIKV HLA class II ligands would be accessible to direct interaction with HLA class II molecules if the denaturative environment of the lysosomal compartment allows first the unfolding of the viral proteins and thus, the non-exposed protein domains could be displayed to HLA class II binding.

The different HLA class I and II molecules analyzed in this study are very common in the different human ethnic groups. For example, HLA-A2 is the human MHC with higher allelic frequency in human population. This allele together with the other HLA class I and II molecules from the two human cell lines analyzed here represent about half of the human population [61], indicating that the data obtained in this study may have great relevance in the study of both CHIKV infection and in the context of the poxvirus-based vaccines that are currently being developed against this emerging pathogen [62,63]. In addition the current report is also relevant about the nature of HLA class II response. So far, it is a common assumption that HLA-DP is less important in the immune response than other HLA class II molecules as -DR or even -DQ proteins, because their lower cell surface expression level [64,65]. However, although for example more than ten thousand T cell epitopes restricted by HLA-DR class II molecules have been reported (Immuno Epitope Database, IEDB; http://www.iedb.org/), a growing body of literature indicates that HLA-DP encodes fully functional molecules, restricting epitope responses in the context of cancer, allergy, and, of course, infectious diseases. Thus, more than a hundred of viral ligands or epitopes restricted by HLA-DP class II molecules have been collected in the IEDB database in the last years. In addition, as a presenting molecule, HLA-DP shows two very interesting features: first, its limited polymorphism regarding HLA-DR locus and second, the existence of a DP supertype that include >90% of the human population [66], where about the three quarters of peptide repertoires are common to four or five HLA-DP class II molecules included in this supertype [66]. In the current report, one and seven CHKV HLA-DR ligands from JY and HOM-2 cells were identified, respectively. In addition, other three CHIKV peptides from HOM-2 cells were also identified as HLA-DPB1*04:01-restricted ligands, allele included in the HLA-DP supertype. Thus, in the antigen presentation by HLA class II of some viral proteins, similar number of HLA-DP vs -DR ligands can be obtained despite the higher cell surface expression of HLA-DR molecules than HLA-DP proteins [64,65].

Overall, this report has identified the CHIKV viral epitopes that bind to different HLA class I and II molecules, as well as those triggered by the recombinant VACV vector, providing an in depth characterization of the repertoire of HLA-restricted epitopes formed during infection of human cells with a vaccine vector, information relevant to understand viral pathogenesis and development of vaccines against pathogens. In addition, the natural CHIKV viral epitopes identified in the current report open a new way of studying more specifically the role of both CD4^+^ and CD8^+^ T lymphocytes in the infection as well as in the chronic pathology caused by CHIKV.

## Supporting information

S1 Fig Identification of the CHIKV HLA class I and II ligands in infected cell extracts by mass spectrometry. MS/MS fragmentation spectra obtained from quadrupole ion trap mass spectrometry of the ion peak indicated in Table 1 from the CHIKV-VACV-infected cell extracts. The vertical axis represents the relative abundance of the parental ion and each fragmentation ion detected. Ions generated in the fragmentation are detailed, and the sequence deduced from the indicated fragments is shown in the upper left side of each panel.
(PDF)

S2 Fig Identification of the CHIKV HLA class I ligand CP_17-25_ by mass spectrometry. MS/MS fragmentation spectra obtained from quadrupole ion trap mass spectrometry of the synthetic RPWTPRPTI peptide corresponding to ion peak at 375.2 from the CHIKV-VACV-infected cell extracts. The vertical axis represents the relative abundance of the parental ion and each fragmentation ion detected. Ions generated in the fragmentation are detailed, and the sequence deduced from the indicated fragments is shown in the upper left side of each panel.
(PDF)

S3 Fig Identification of the CHIKV HLA class II ligand E2_133-145_ by mass spectrometry. MS/MS fragmentation spectra obtained from quadrupole ion trap mass spectrometry of the synthetic PPVIGREKFHSRP peptide corresponding to ion peak at 380.7 from the CHIKV-VACV-infected cell extracts. The vertical axis represents the relative abundance of the parental ion and each fragmentation ion detected. Ions generated in the fragmentation are detailed, and the sequence deduced from the indicated fragments is shown in the upper left side of each panel.
(PDF)
