# Supplementary figures and images for: Immunoproteomic analysis of a Chikungunya poxvirus-based vaccine reveals high HLA class II immunoprevalence
Source: PLoS Negl Trop Dis. 2019 Jul 5;13(7):e0007547. doi: 10.1371/journal.pntd.0007547 (PMC6636782; doi:10.1371/journal.pntd.0007547)

R P W T P R P T I

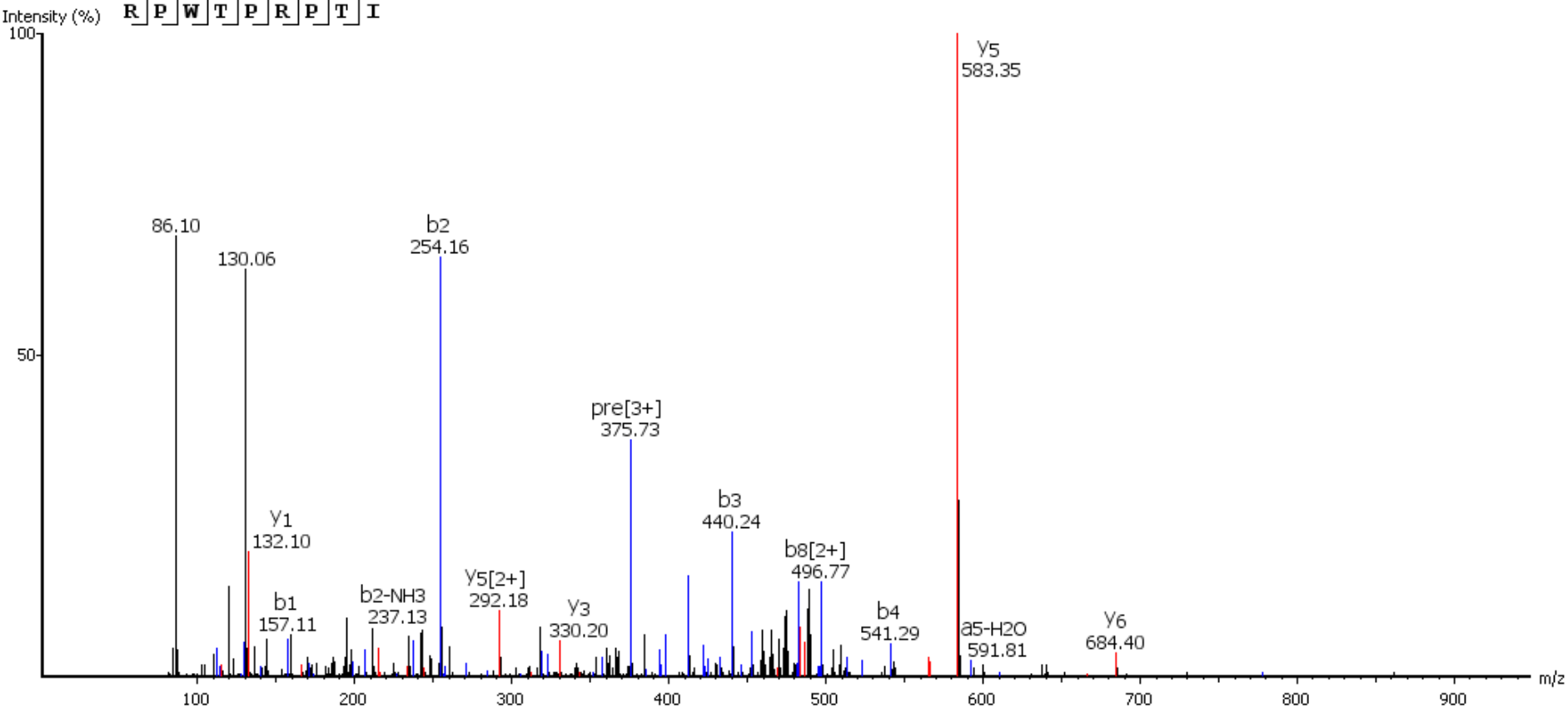

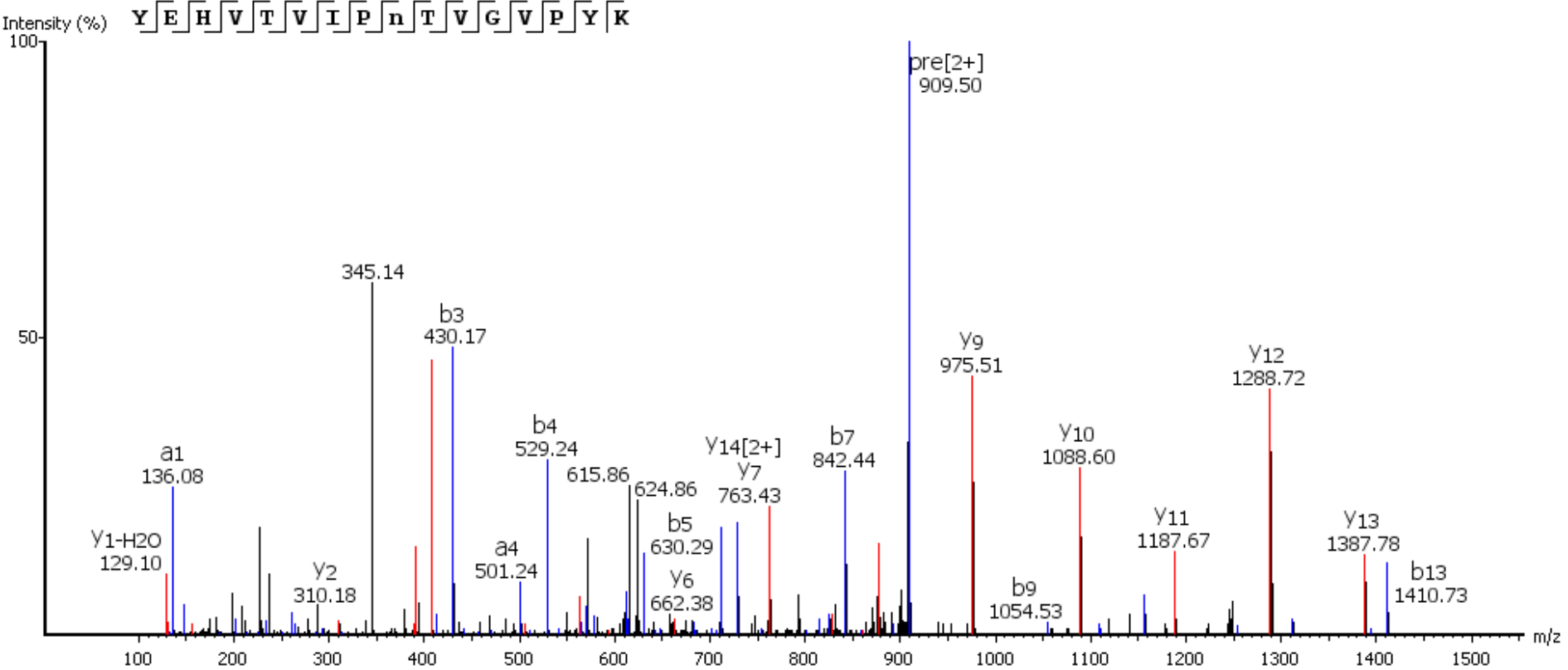

Intensity (%) **Y E H V T V I P N T V G V P Y K**

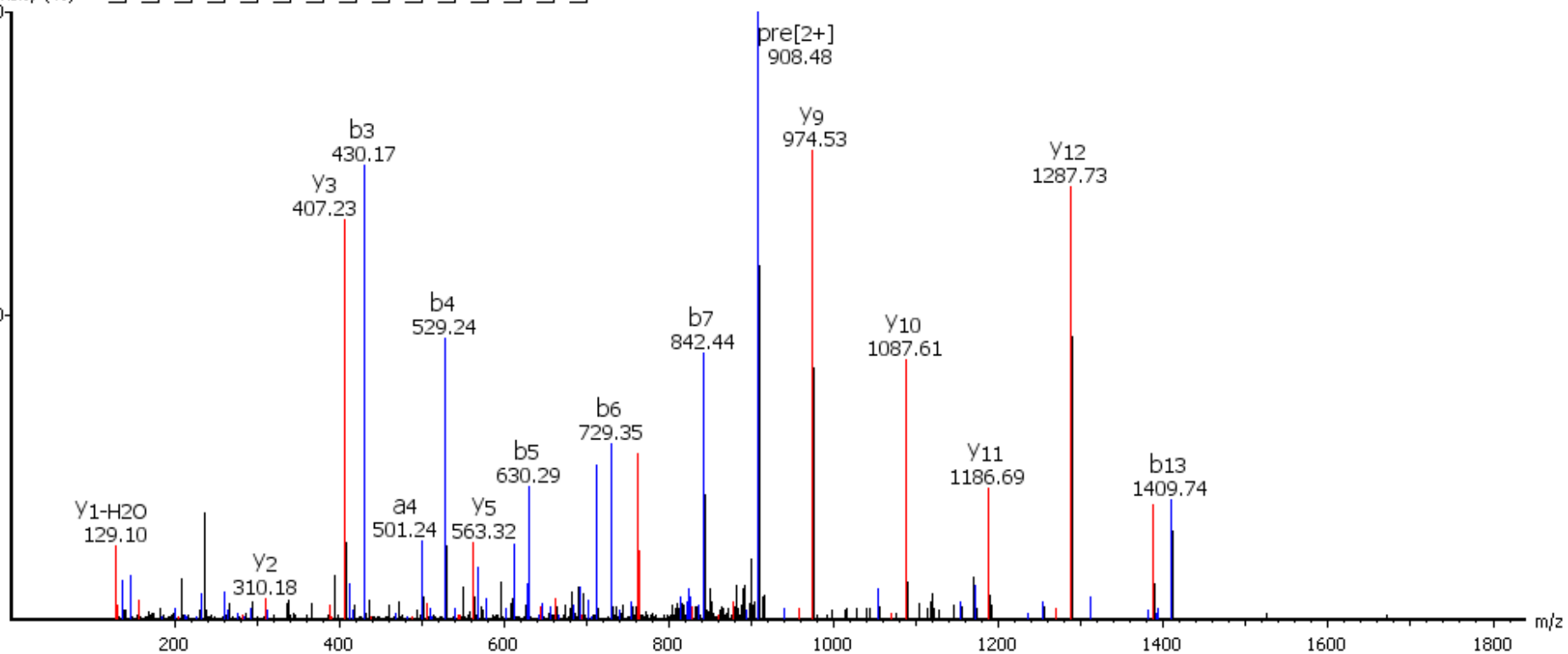

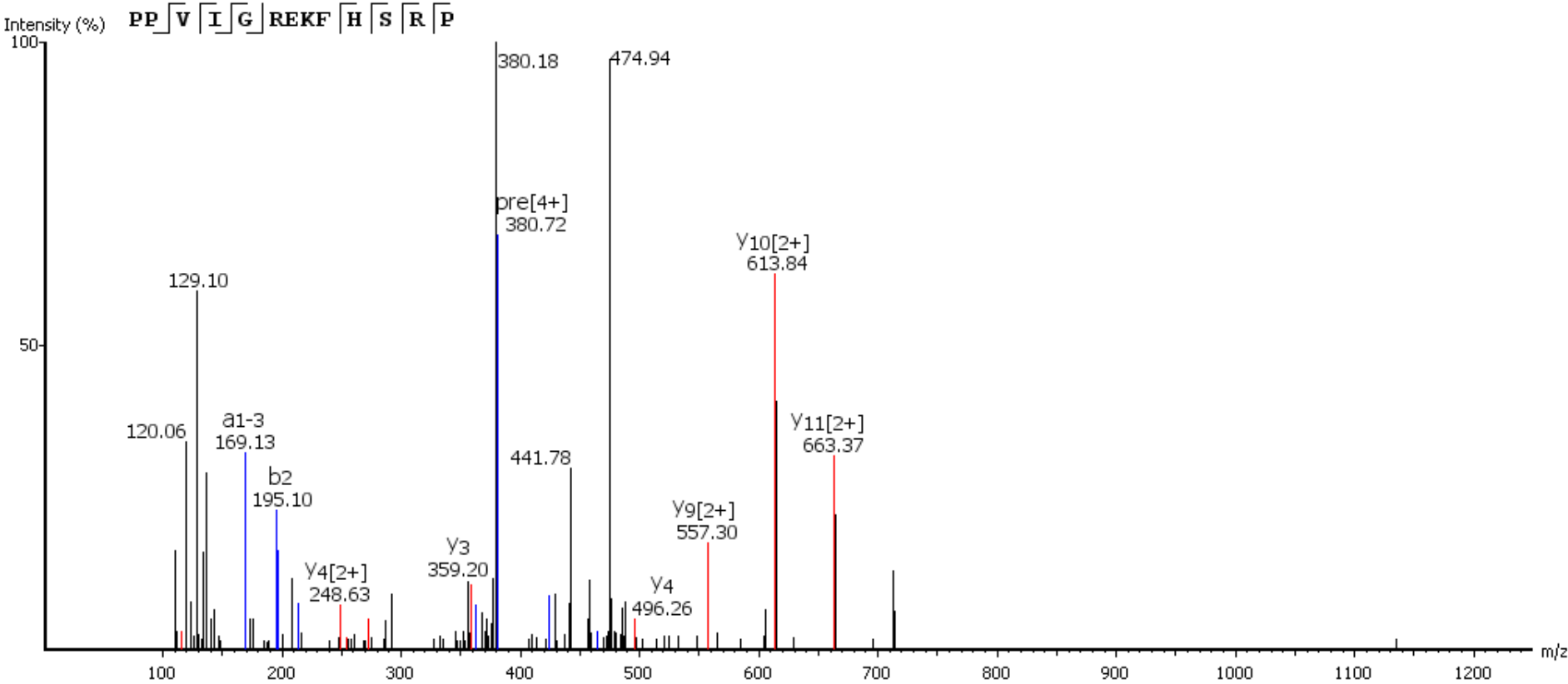

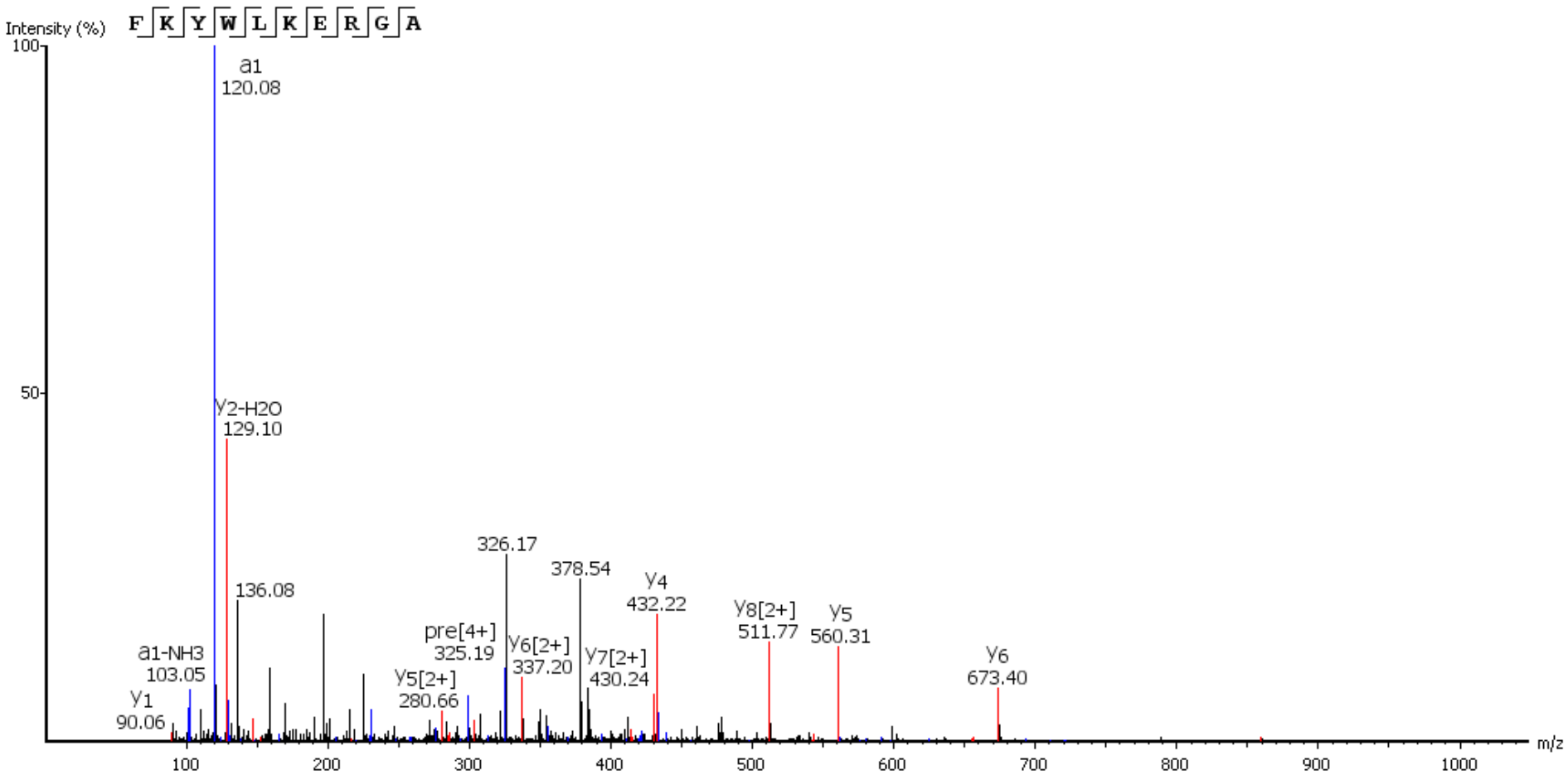

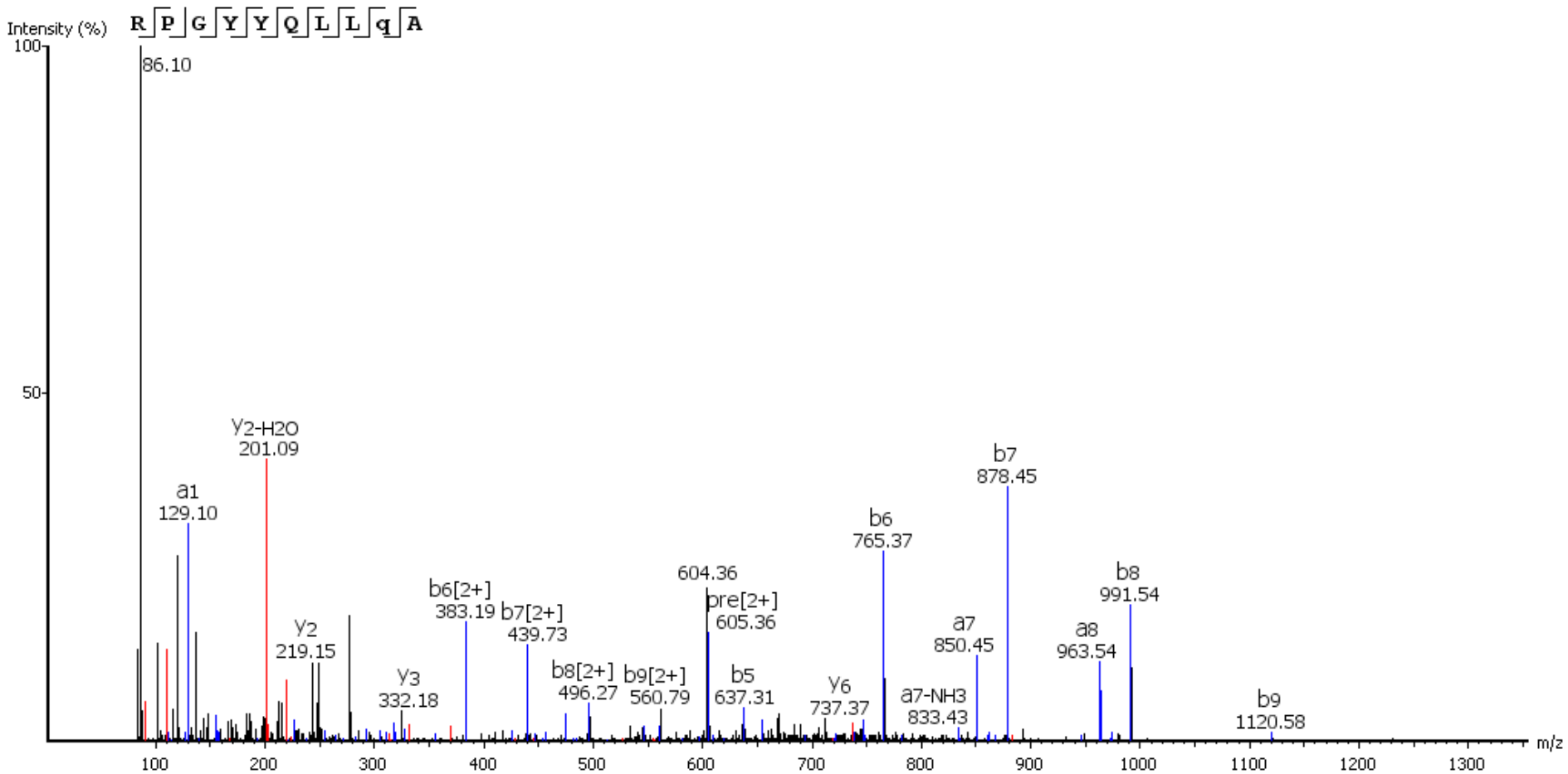

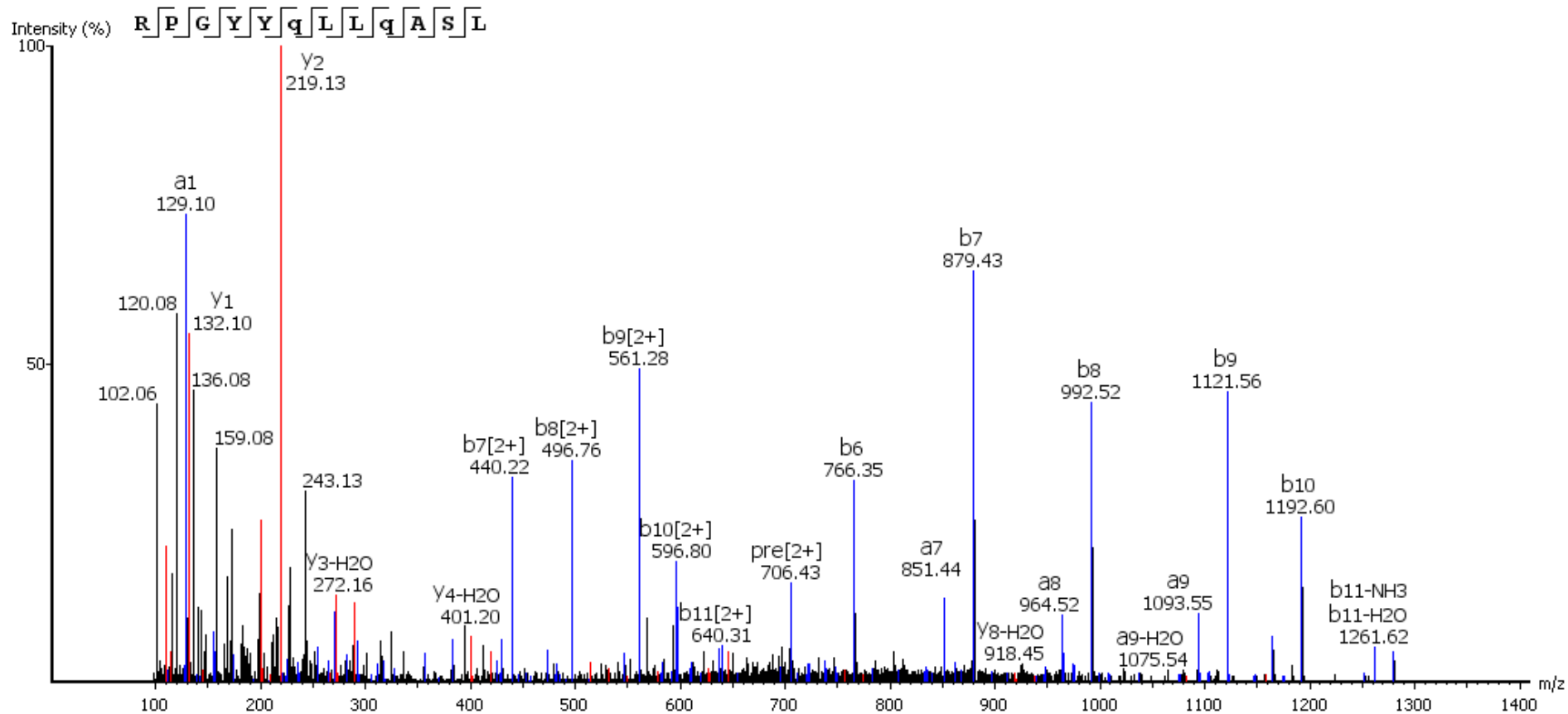

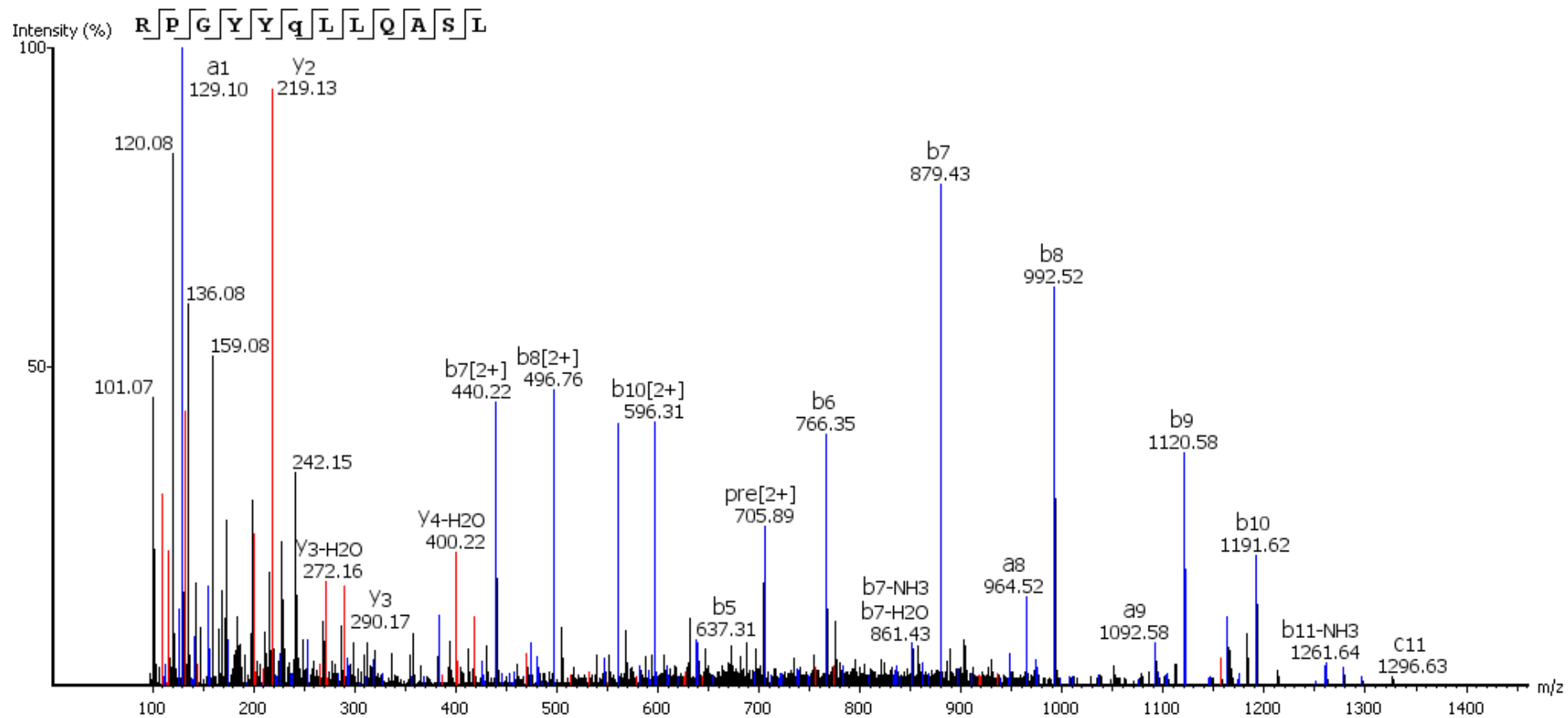

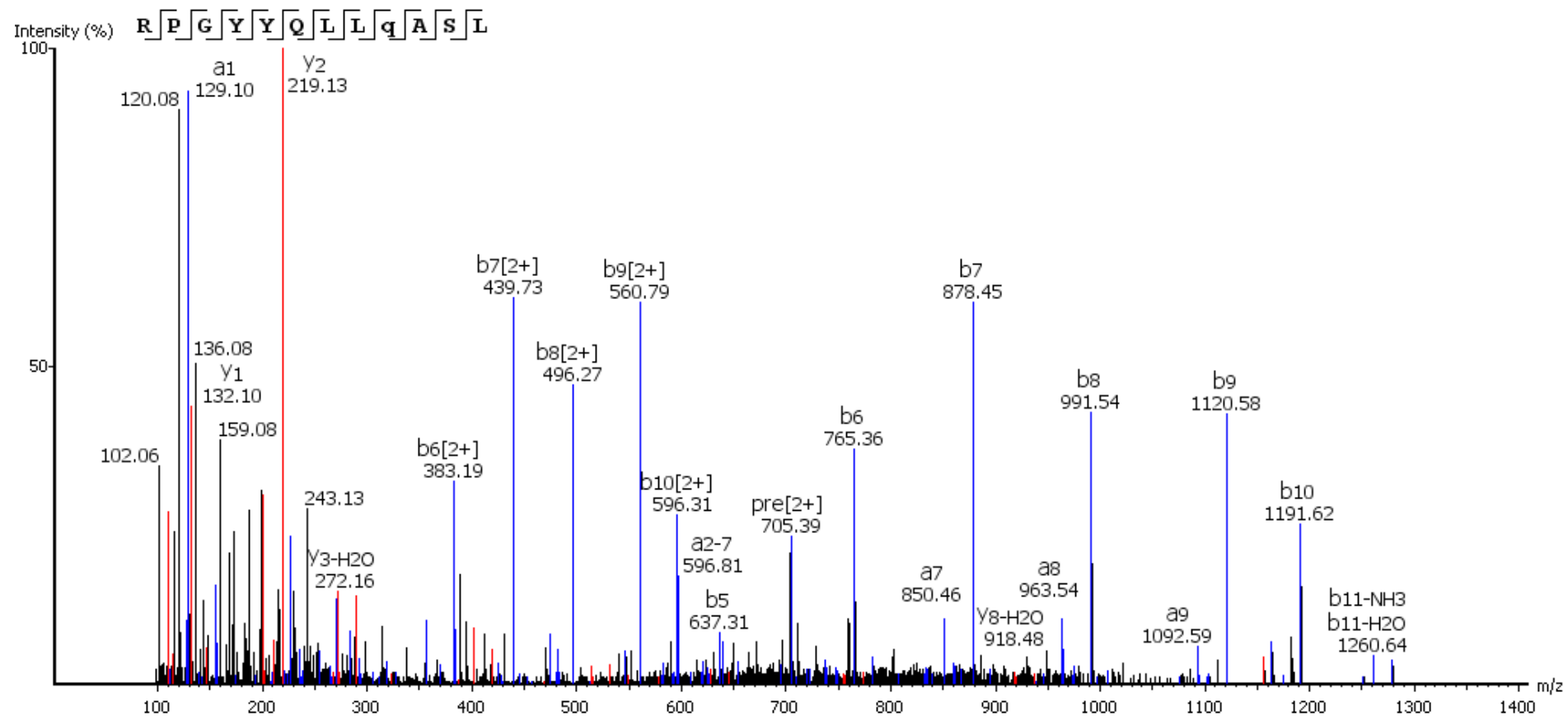

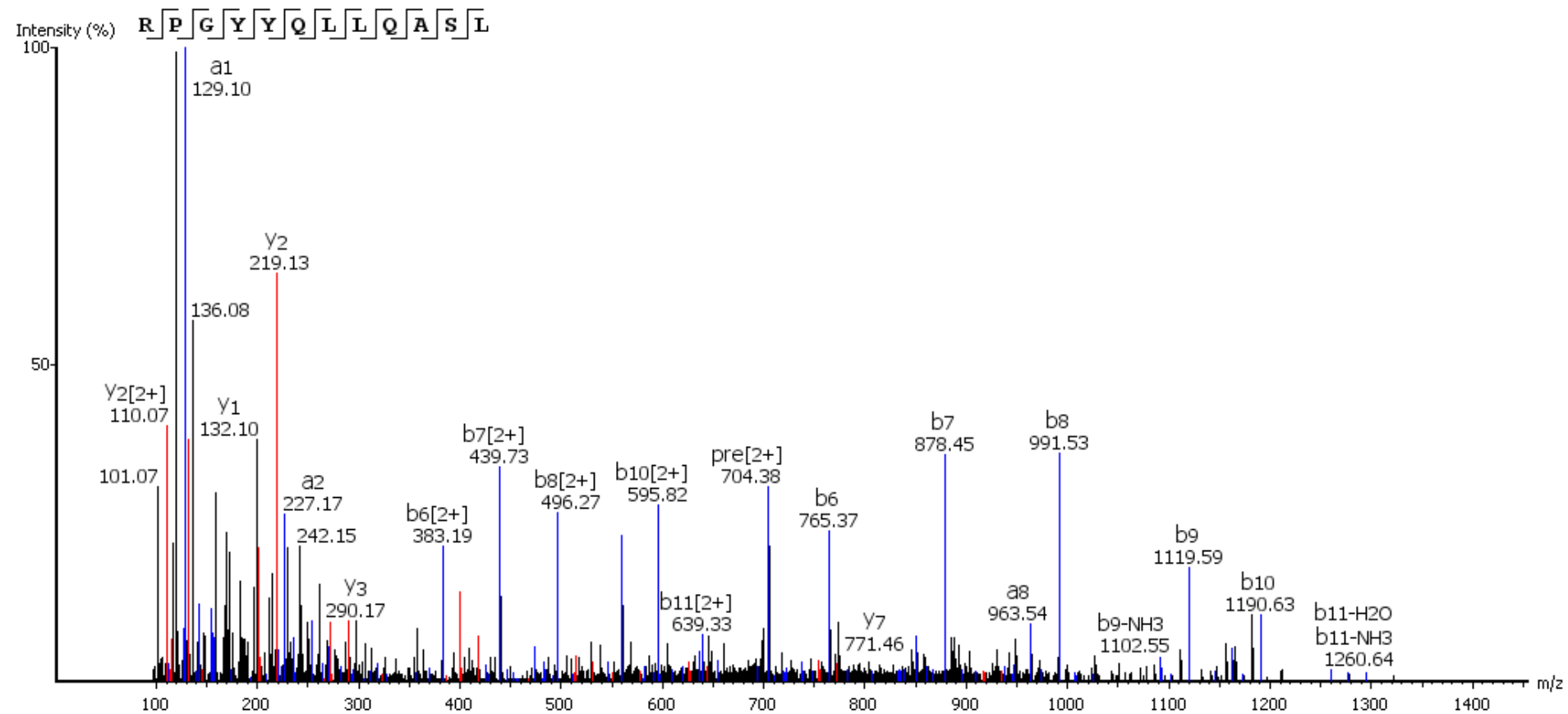

RPGYYQLLQASL

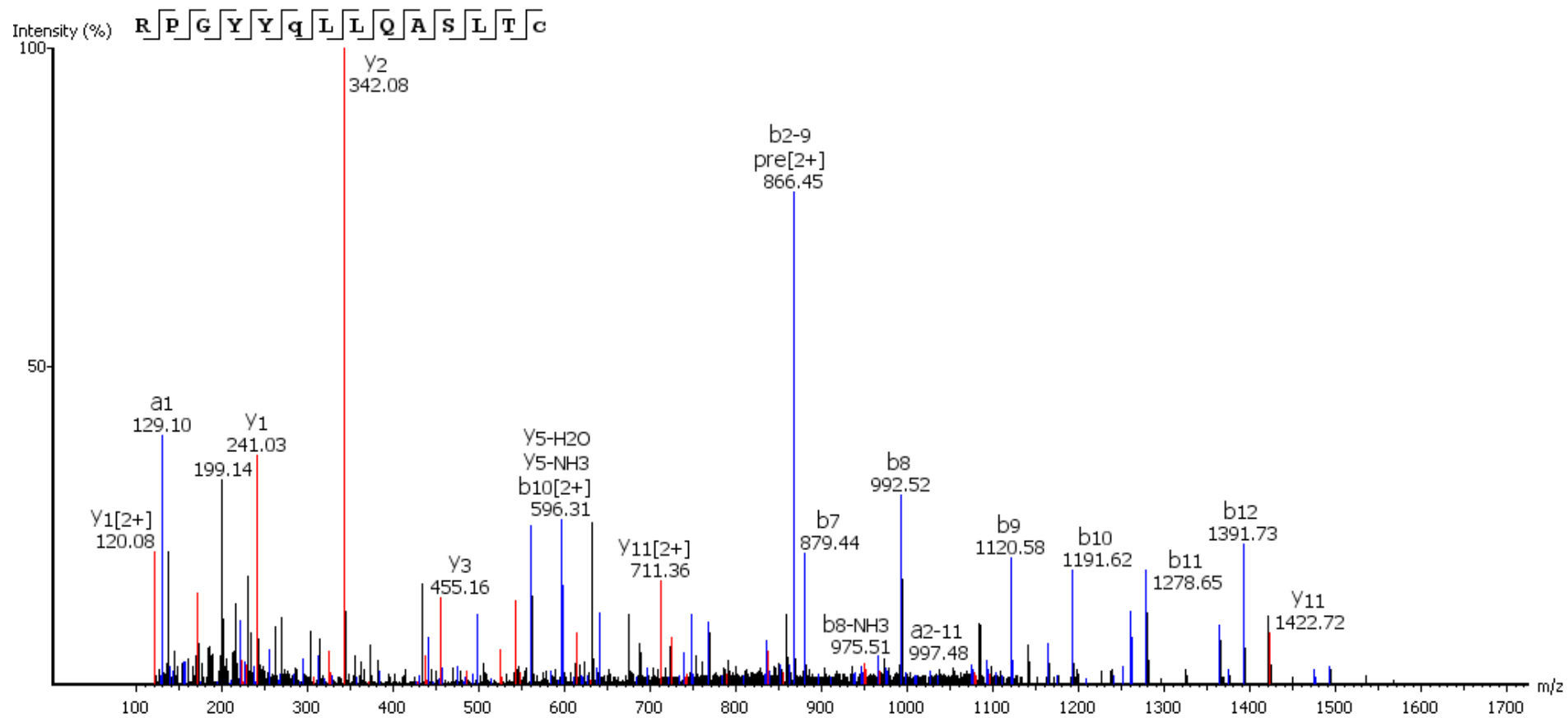

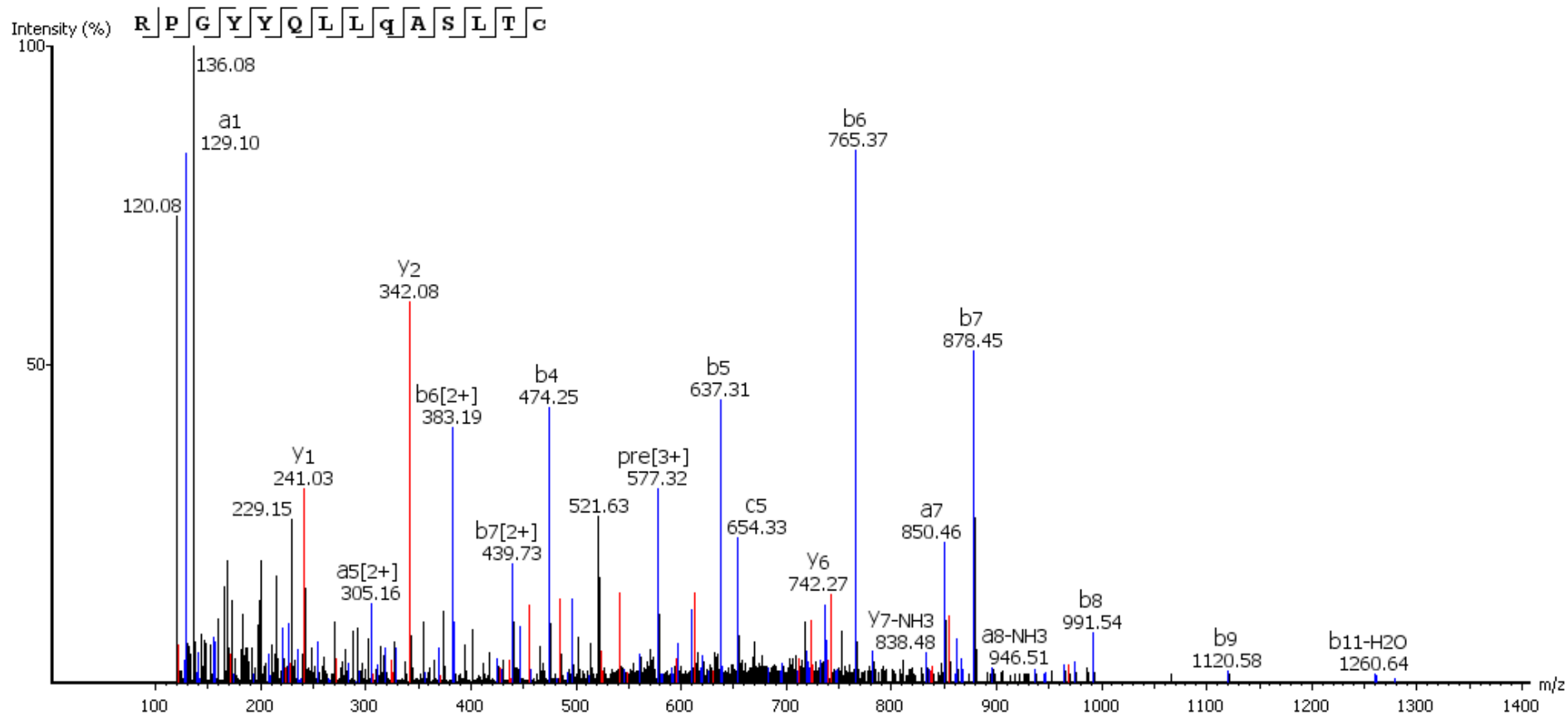

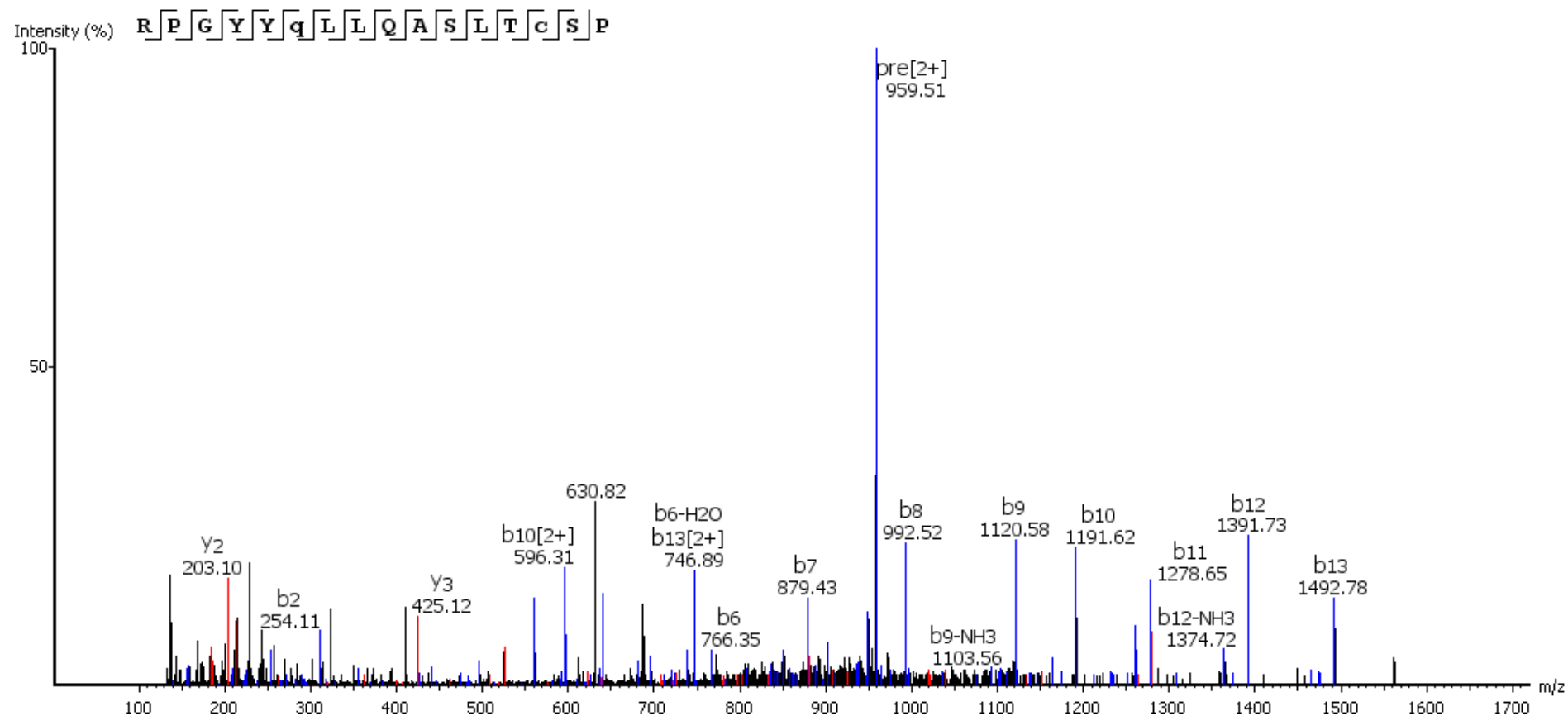

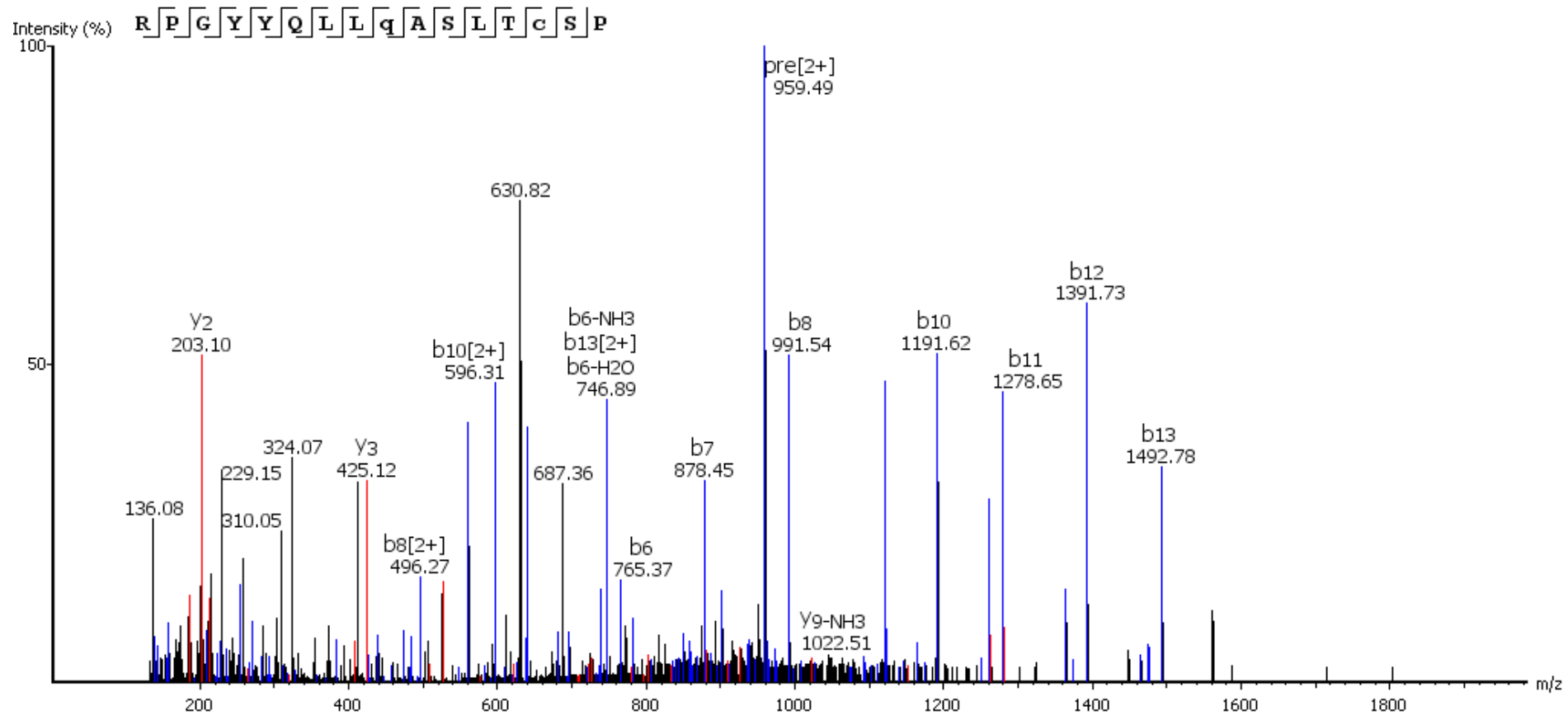

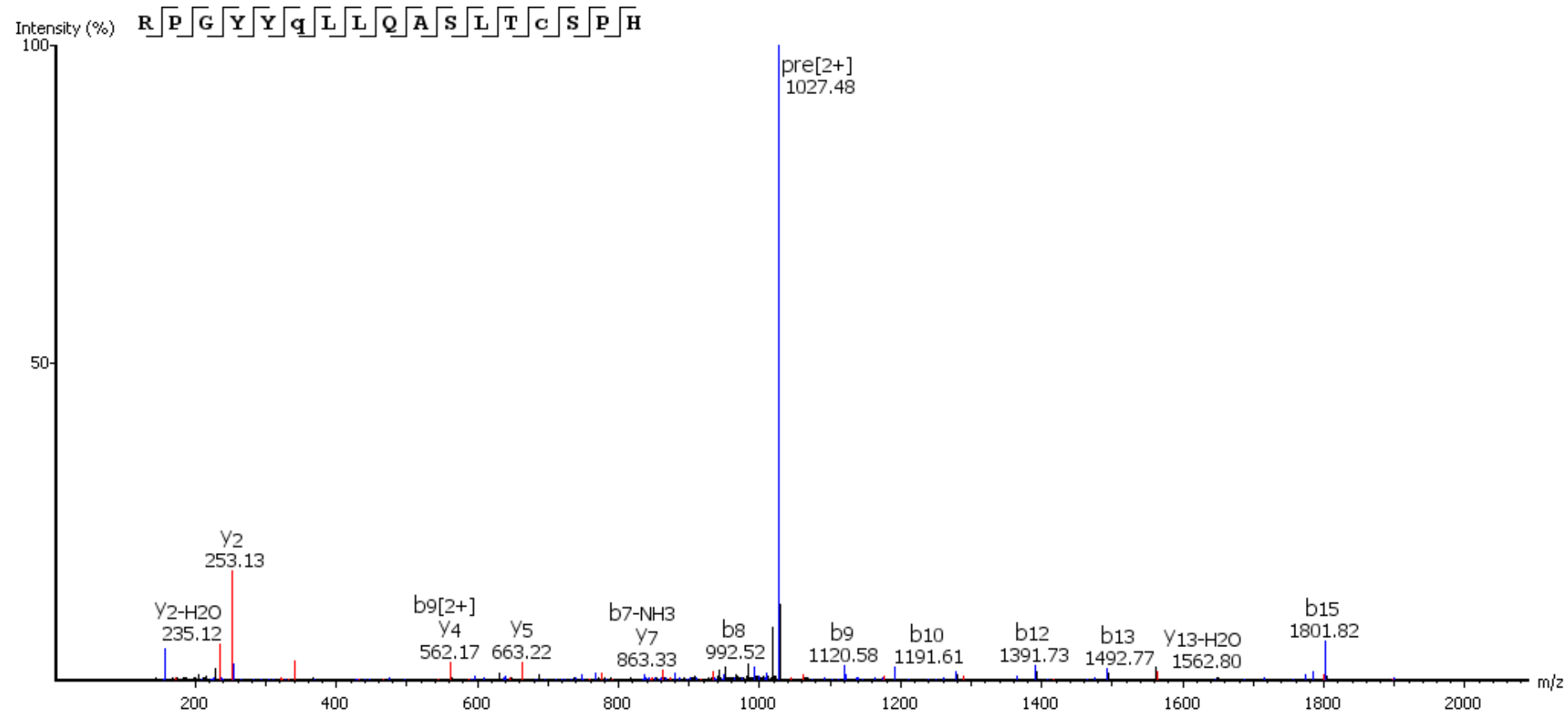

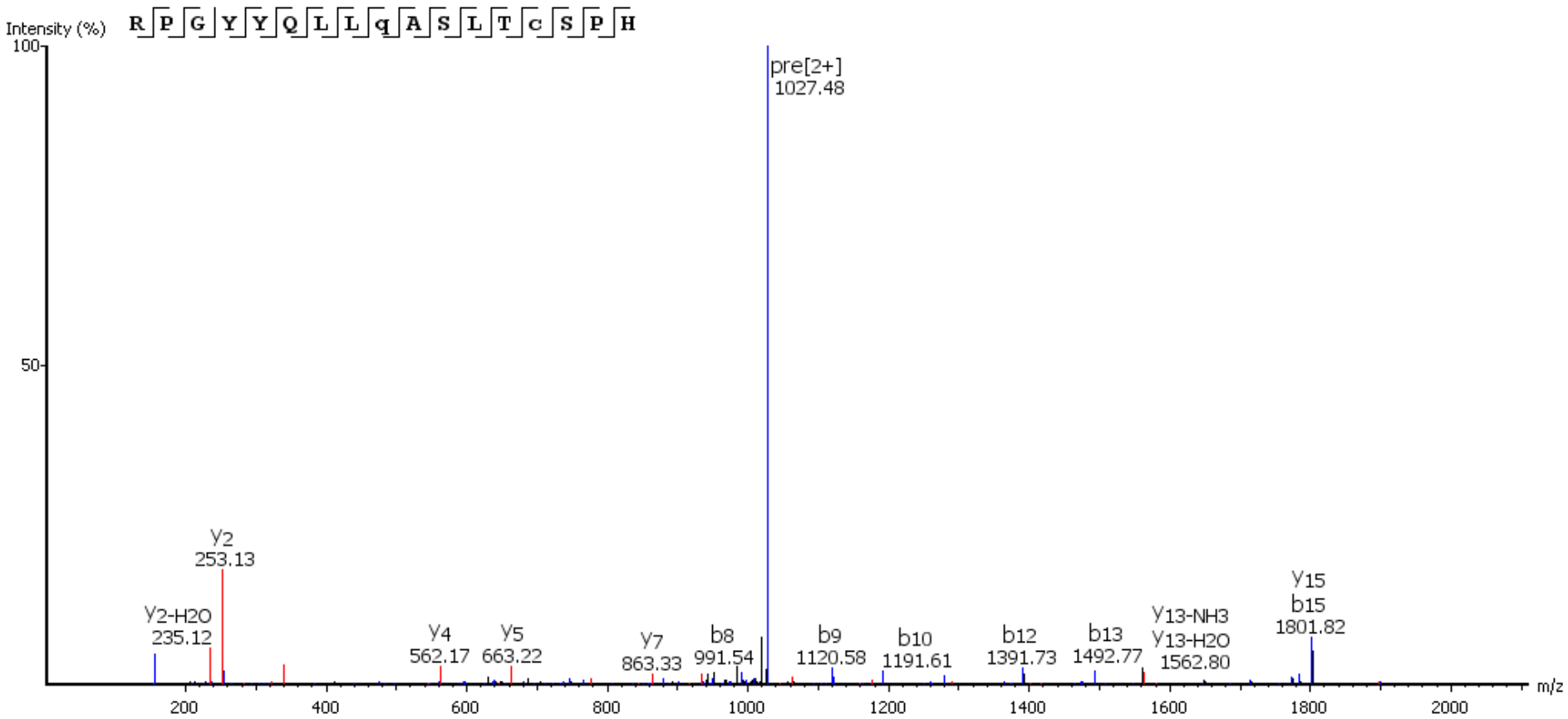

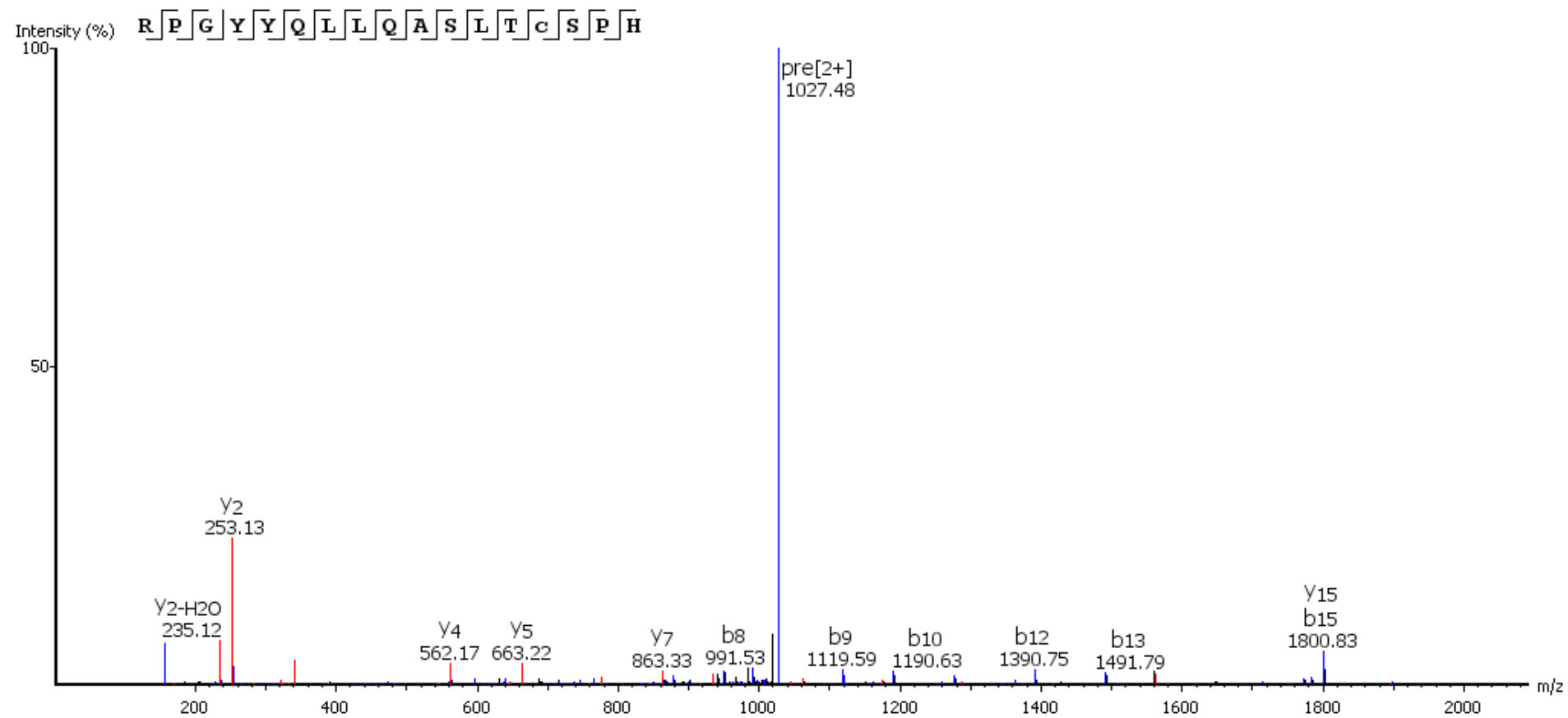

R P G Y Y Q L L Q A S L T C S P H R

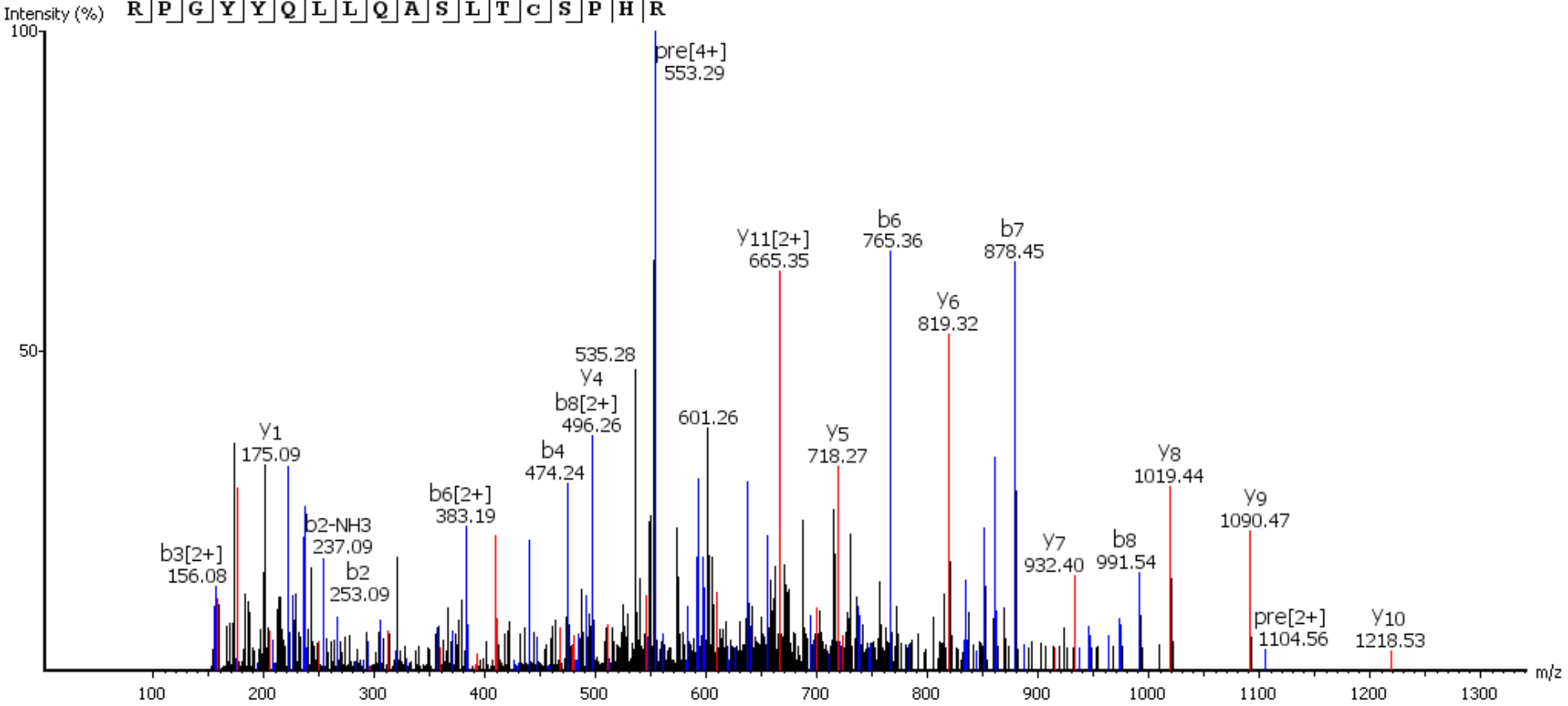

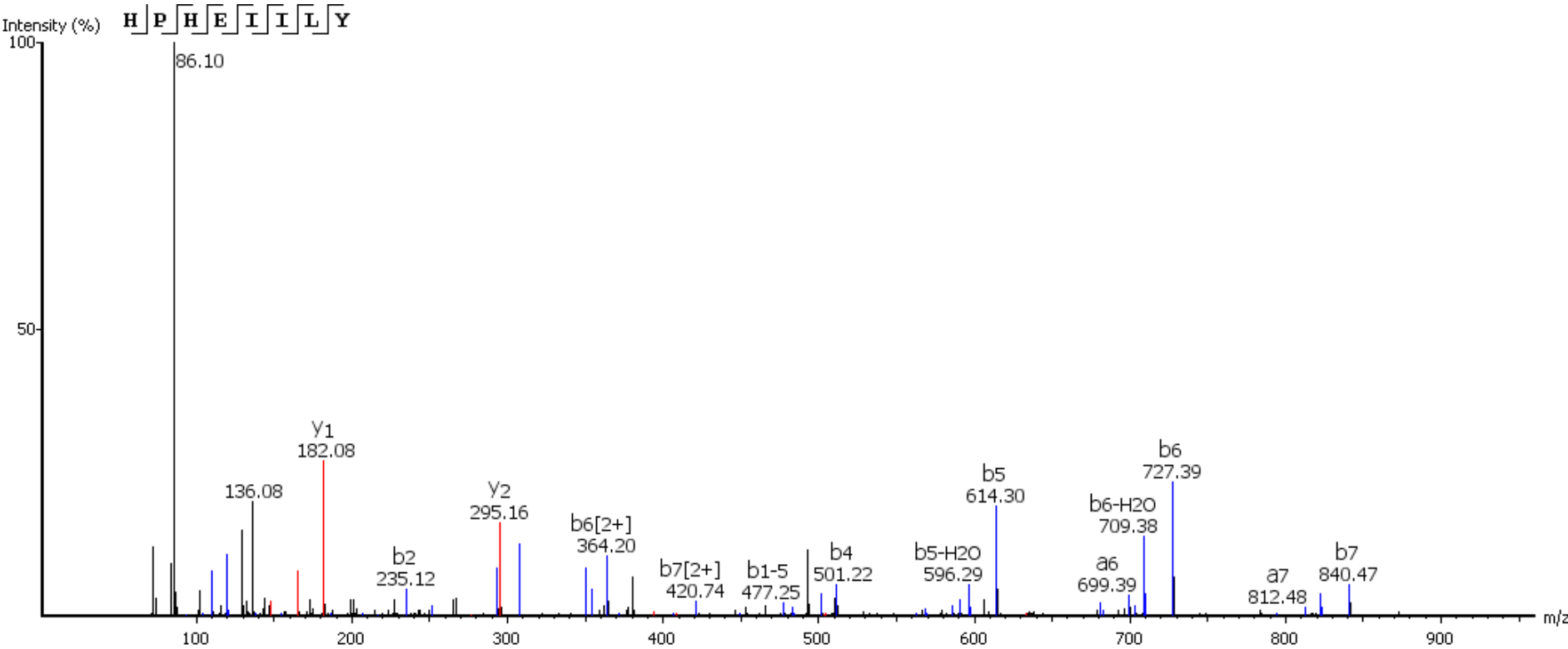

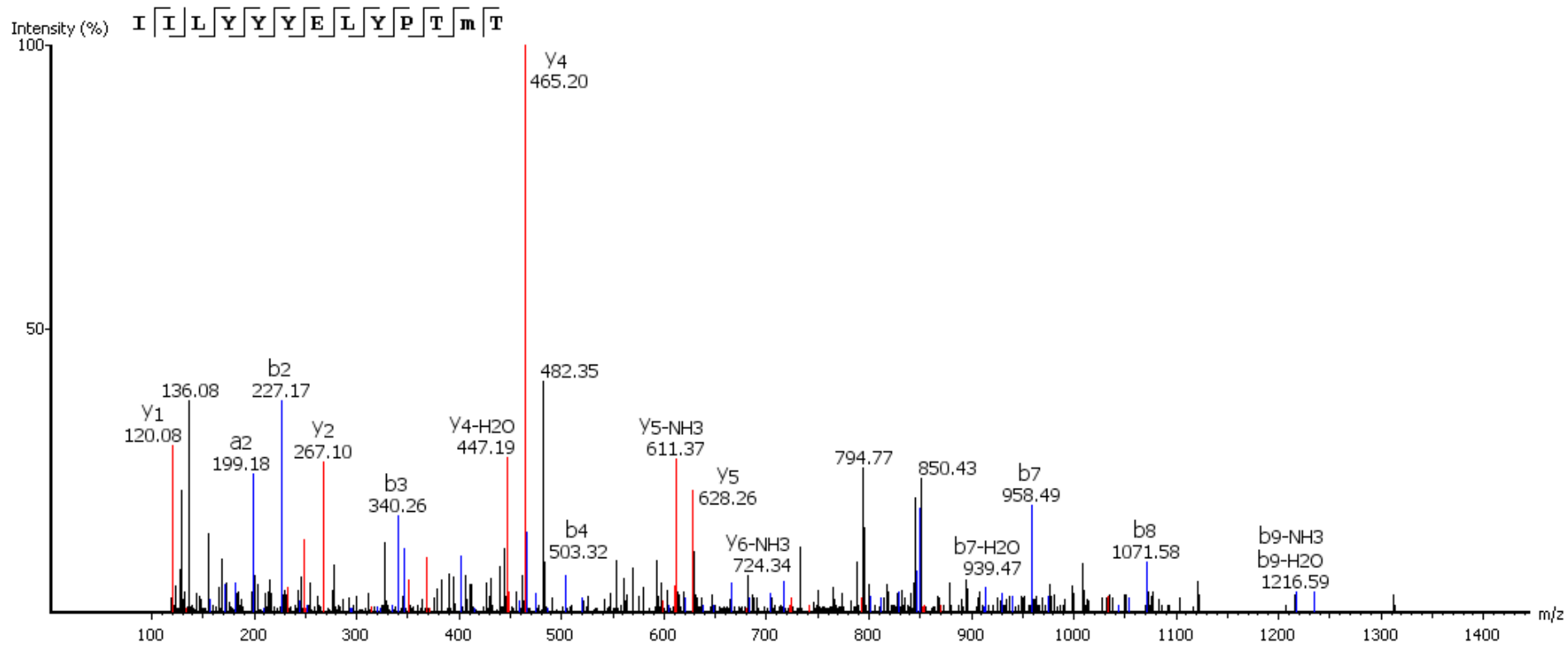

Supplement: S1 Fig — MS/MS fragmentation spectra obtained from quadrupole ion trap mass spectrometry of the ion peak indicated in Table 1 from the CHIKV-VACV-infected cell extracts. The vertical axis represents the relative abundance of the parental ion and each fragmentation ion detected. Ions generated in the fragmentation are detailed, and the sequence deduced from the indicated fragments is shown in the upper left side of each panel. (PDF) [file pntd.0007547.s001.pdf]

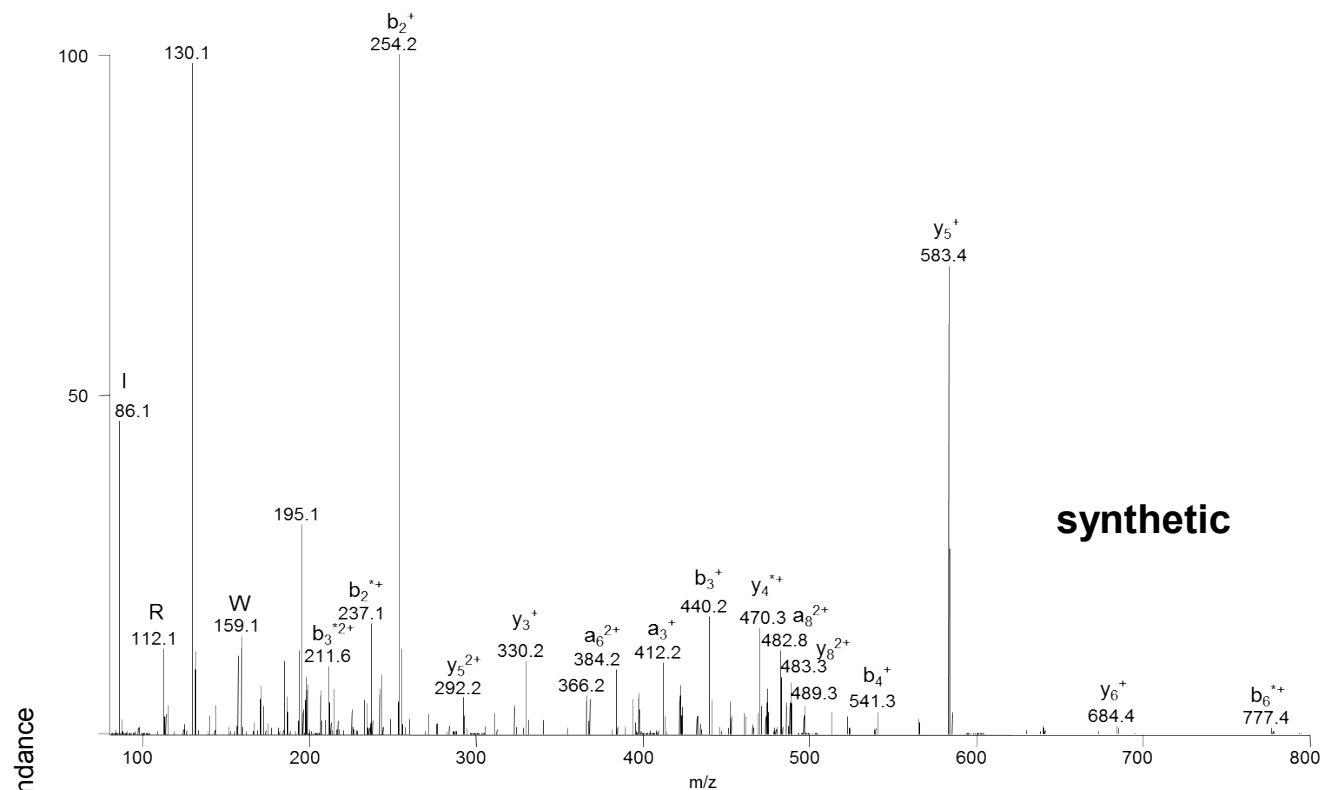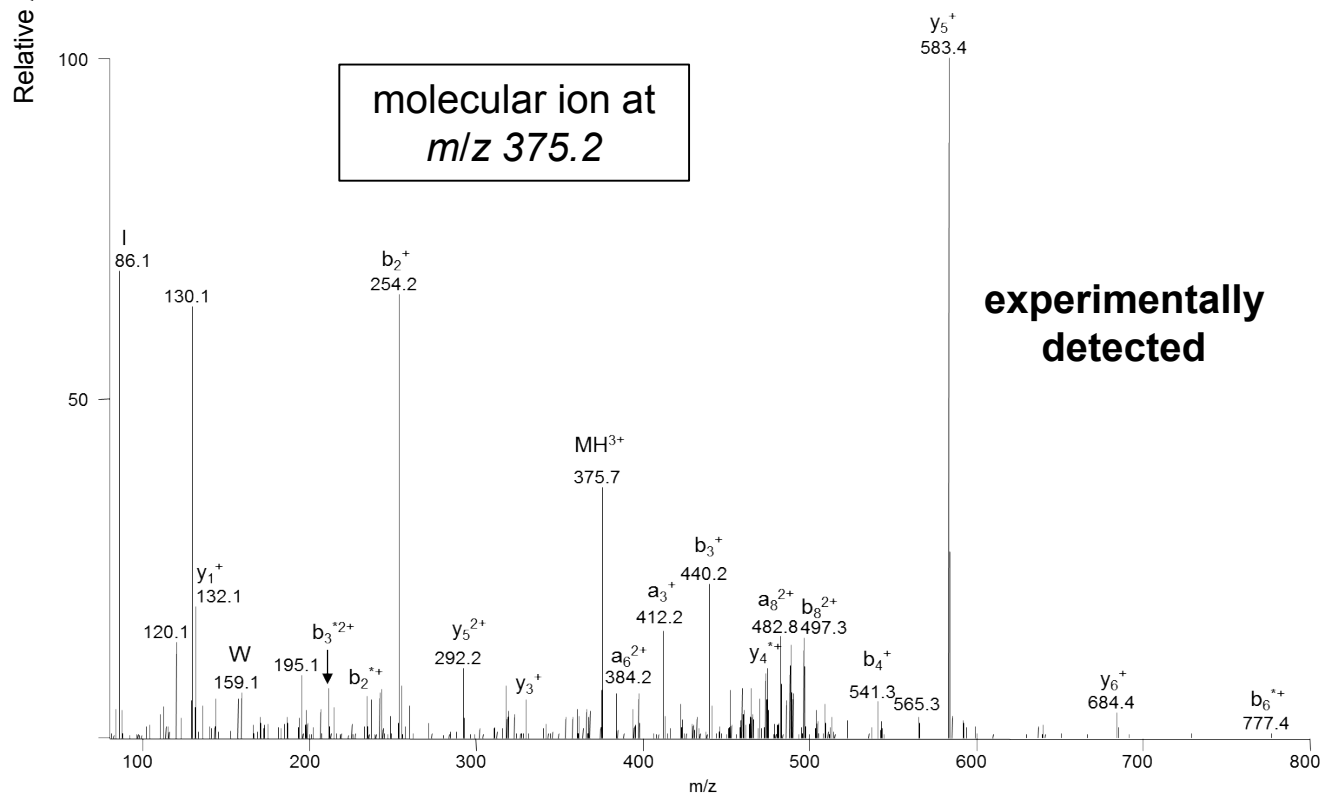

RPWTPRPTI

Supplementary Figure 1

Supplement: S2 Fig — MS/MS fragmentation spectra obtained from quadrupole ion trap mass spectrometry of the synthetic RPWTPRPTI peptide corresponding to ion peak at 375.2 from the CHIKV-VACV-infected cell extracts. The vertical axis represents the relative abundance of the parental ion and each fragmentation ion detected. Ions generated in the fragmentation are detailed, and the sequence deduced from the indicated fragments is shown in the upper left side of each panel. (PDF) [file pntd.0007547.s002.pdf]

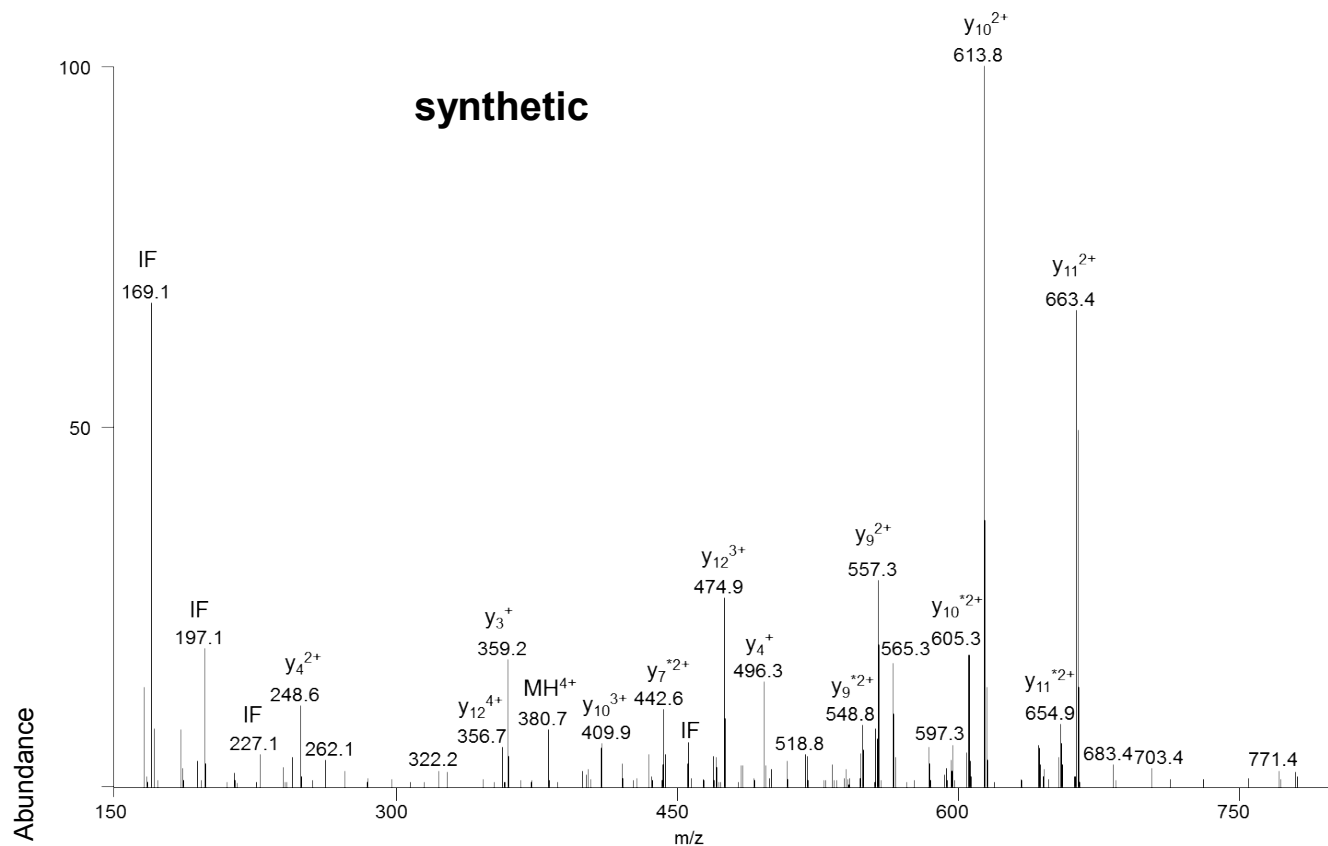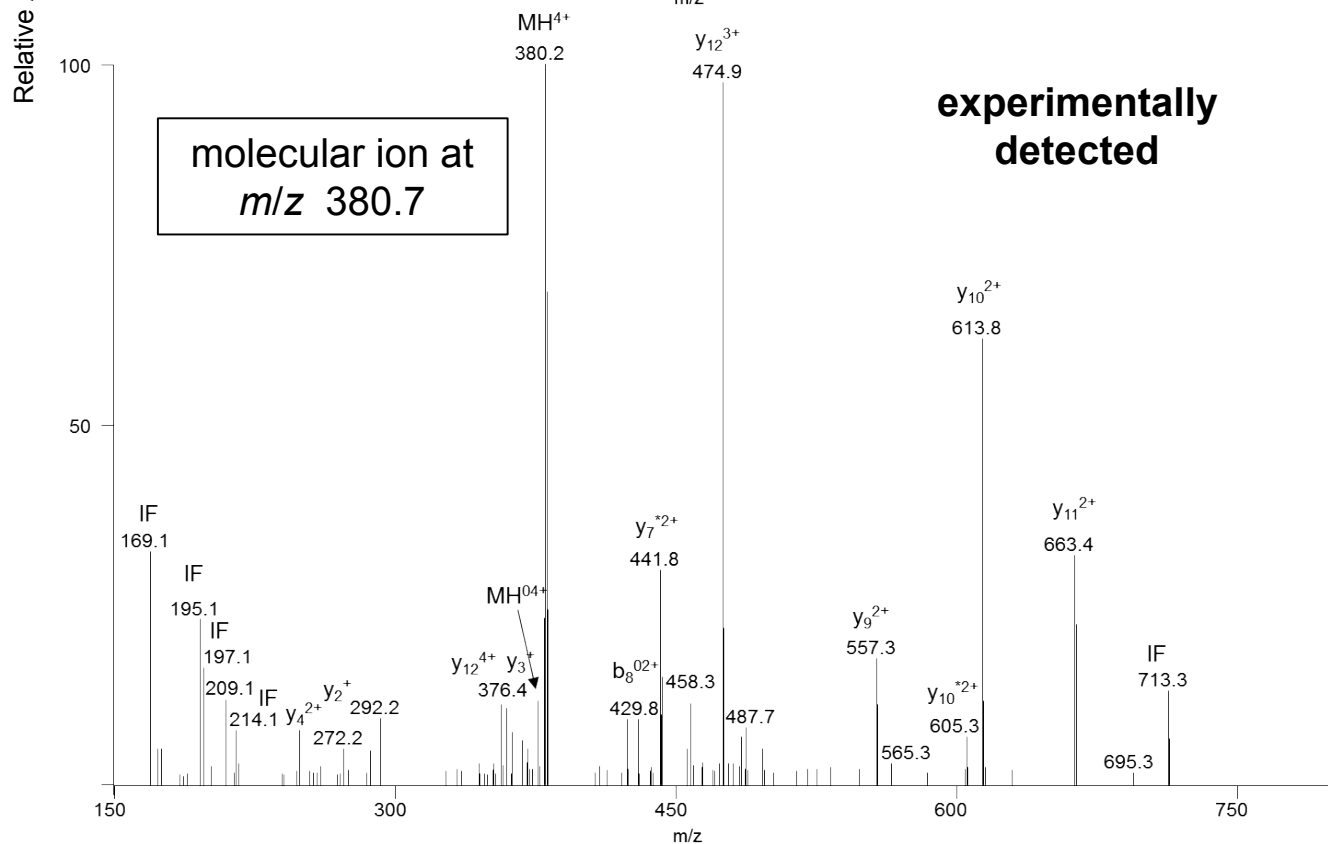

PPVIGREKFHSRP

Supplementary Figure 2

Supplement: S3 Fig — MS/MS fragmentation spectra obtained from quadrupole ion trap mass spectrometry of the synthetic PPVIGREKFHSRP peptide corresponding to ion peak at 380.7 from the CHIKV-VACV-infected cell extracts. The vertical axis represents the relative abundance of the parental ion and each fragmentation ion detected. Ions generated in the fragmentation are detailed, and the sequence deduced from the indicated fragments is shown in the upper left side of each panel. (PDF) [file pntd.0007547.s003.pdf]
